# Development and Characterization of High-Throughput EST-Based SSR Markers for *Pogostemon cablin* Using Transcriptome Sequencing

**DOI:** 10.3390/molecules23082014

**Published:** 2018-08-13

**Authors:** Puyue Ouyang, Dali Kang, Xiaolu Mo, Enwei Tian, Yanyu Hu, Rongshao Huang

**Affiliations:** 1 Agricultural College, Guangxi University, Nanning 530005, China; ouyangpy@gdyzy.edu.cn (P.O.); striverhu@163.com (Y.H.); 2 College of Chinese Materia Medica, Guangdong Food and Drug Vocational College, Guangzhou 510520, China; moxl@gdyzy.edu.cn; 3 College of Medical Technology, Ningbo College of Health Sciences, Ningbo 315100, China; 202099@nchs.edu.cn; 4 School of Traditional Chinese Medicine, Southern Medical University, Guangzhou 510515, China; tianenwei@126.com

**Keywords:** *Pogostemon cablin*, expressed sequence tag (EST), simple sequence repeat (SSR), polymorphism, transferability

## Abstract

Simple sequence repeats (SSRs) or microsatellite markers derived from expressed sequence tags (ESTs) are routinely used for molecular assisted-selection breeding, comparative genomic analysis, and genetic diversity studies. In this study, we investigated 54,546 ESTs for the identification and development of SSR markers in *Pogostemon cablin* (Patchouli). In total, 1219 SSRs were identified from 1144 SSR-containing ESTs. Trinucleotides (80.8%) were the most abundant SSRs, followed by di- (10.8%), mono- (7.1%), and hexa-nucleotides (1.3%). The top six motifs were CCG/CGG (15.3%), AAG/CTT (15.0%), ACC/GGT (13.5%), AGG/CCT (12.4%), ATC/ATG (9.9%), and AG/CT (9.8%). On the basis of these SSR-containing ESTs, a total of 192 primer pairs were randomly designed and used for polymorphism analysis in 38 accessions collected from different geographical regions of Guangdong, China. Of the SSR markers, 45 were polymorphic and had allele variations from two to four. Furthermore, a transferability analysis of these primer pairs revealed a 10–40% cross-species transferability in 10 related species. This report is the first comprehensive study on the development and analysis of a large set of SSR markers in *P. cablin*. These markers have the potential to be used in quantitative trait loci mapping, genetic diversity studies, and the fingerprinting of cultivars of *P. cablin.*

## 1. Introduction

*Pogostemon cablin* (Blanco) Benth., Laminaceae (Patchouli) is a hardy, perennial, and aromatic herb that adapts to hot and humid climatic surroundings. It is widely distributed in the wilds of Indonesia, Philippines, Malaysia, and other countries of Southeast Asia, and is extensively cultivated in South China [1]. Patchouli essential oil is one of the most important materials used in the perfume industry [2]. In China, *P. cablin* is also used as a medicine for the treatment of the common cold, headache, fever, vomiting, indigestion, and diarrhea [3,4]. Patchouli oil is also known for its protection against gastric ulcers [5] and its antiseptic, anti-inflammatory, and antifungal properties [6,7]. Nowadays, growing interest in its fragrance and medicinal applications has promoted patchouli’s cultivation in South China. Nevertheless, the therapeutic properties of *P. cablin* are directly correlated with the qualitative and quantitative composition of its components, such as patchouli oil, which are obviously different depending on the cultivar region of origin [8].

In this genomic era, deciphering the genomes of medicinal plants will enable insights into the biochemistry of the genes responsible for secondary metabolism biosynthetic pathways, and then improve the contents of the active ingredients. Recent advances in genome sequencing technologies and assembly methods have resulted in the generation of genome sequences for a wide range of plant species. However, despite the prominent roles of *P. cablin* in both traditional Chinese medicine and the perfume industry, very few studies have examined the genomes of *P. cablin*. He et al. [9] reported the first de novo assembled 1.15-Gb draft genome sequence for *P. cablin* from next-generation sequencing technology in 2016, which is also the first draft of the genome of Lamiaceae.

In modern phylogenetic studies and genetic breeding studies of plant cultivars, molecular markers have become important and efficient tools for genetic diversity assessment, quantitative trait loci (QTL) analysis, and gene mapping. The genetic improvement strategy mainly relies on access to genetic variation in the gene pool [10]. Diverse genetic materials need to be assessed for genetic diversity among the accessions collected from widely separated geographical locations. Molecular phylogenetic studies have clarified many relationships within Lamiaceae, inferred from cpDNA sequences, nuclear DNA, and plastid DNA [11,12,13,14,15,16,17], but the genus *Pogostemon* has received relatively scant attention. Some efforts have been made to assess the genetic variability in *P. cablin*; however, only a few molecular marker-based studies have been carried out to achieve this goal, employing, for instance, the random amplification of polymorphic DNA (RAPD) [18,19], inter-simple sequence repeat (ISSR), and sequence-related amplified polymorphism (SRAP) [20]. These studies showed different levels of polymorphism in *P. cablin*, suggesting the necessity to develop a large number of markers that can be utilized for germplasm characterization and QTL analysis for *P. cablin*. 

Simple sequence repeats (SSRs) or microsatellites consist of short tandem repeated motifs of 1 to 6 bp units, and occur in both the coding and noncoding regions of the genome [21,22]. SSR markers are a good choice for a broad application in basic and applied plant biology, because of their high level of polymorphism, high abundance, co-dominance, reproducibility, and high rates of transferability across species and genera [21,23,24]. Expressed sequence tag-simple sequence repeats (EST-SSRs) are markers mined from EST sequence collections. As markers, they differ from traditional genomic SSR (gSSR) markers, as they are more likely embedded in functional gene sequences, less costly to identify, and may provide more abundant information. EST-SSR markers are present in more conserved regions in comparison to markers that are generated from genomic sequences, therefore, they show more transferability between species [24,25]. In this study, we examined 54,546 EST sequences obtained from the transcriptome sequencing of *P. cablin* in order to (1) develop EST-derived SSRs for *P. cablin*; (2) analyze the frequency and distribution of EST-SSRs in the expressed portion of the genome; (3) construct a functional annotation and prediction of amino acids from SSR loci; and (4) evaluate the polymorphism and cross-species and genera transferability of EST-SSR markers.

## 2. Results

### 2.1. Characterization of EST-SSRs 

A total of 54,546 patchouli EST sequences were screened for SSRs. The SSR screening resulted in the identification of 1114 sequences containing 1219 SSRs (Table 1). A total of 1047 sequences contained single SSRs of different motif types, and 67 sequences contained more than two SSRs.

**Figure 1:**
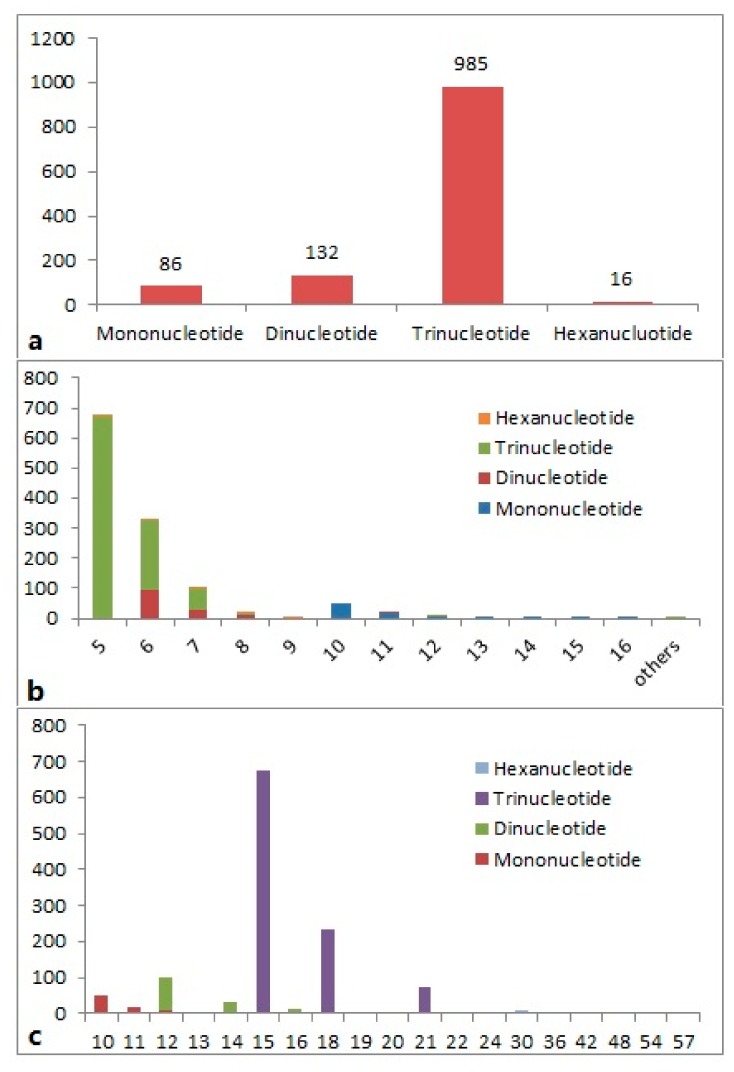
Frequency distribution of the putative expressed sequence tag-simple sequence repeats (EST-SSRs) from patchouli expressed sequence tags (ESTs) based on (**a**) the distribution of EST-SSRs types, (**b**) the EST-SSRs repeat number, and (**c**) the EST-SSRs’ repeat lengths (bp).

Of the total 1219 SSRs, 1182 (97.0%) were simple repeat motifs and 37 (3.0%) were in compound formation. The highest proportion of repeats (80.8%) was represented by trinucleotide repeats, followed by dinucleotide (10.8%) and mononucleotide (7.1%) repeats, and only 1.3% hexanucleotide SSRs were found (Figure 1a). The trinucleotide and dinucleotide repeats thus represented the majority of EST-SSRs in *P. cablin*. The number of repeats of the different SSR motifs varied from 5 to 24, with a maximum frequency for five repeats, followed by six and seven repeats. The frequency of the trinucleotide motifs with five repeats was more common (55.1%), followed by six-repeat trinucleotides (19.2%) and six-repeat dinucleotides (7.6%). The frequency of different SSR motifs for each repeat number is presented in Figure 1b. The length of the SSR region (motif length × repeat numbers) varied from 10 to 57 bases, with a maximum frequency for 15 bases (55.1%), followed by 18 bases (19.2%) and 12 bases (7.6%) (Figure 1c). 

Among the dinucleotide repeats, AG/CT was the most common (90.9%) motif, followed by AC/GT (8.3%) and AT/AT (0.8%). Among the trinucleotide repeats, CCG/CGG was the most common, accounting for 18.9% of the total trinucleotide repeats, followed by the AAG/CTT (18.6%) and ACC/GGT (16.8%) motifs (Table 2). 

### 2.2. Categories and Annotations of Unigenes

**Figure 2:**
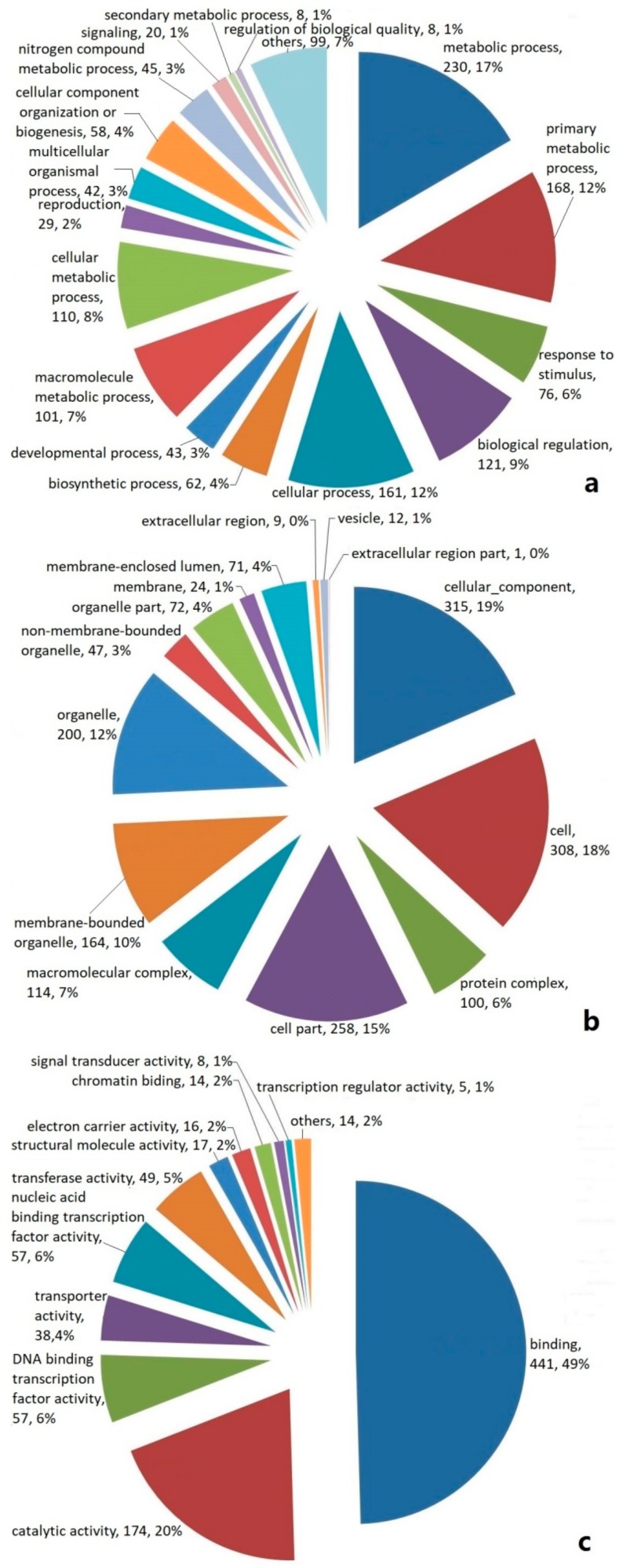
Distribution of EST-SSR-encoded unigenes in *P. cablin* classified according to their biological function, namely, (**a**) biological process, (**b**) cellular component, and (**c**) molecular function.

Based on a Basic Local Alignment Search Tool X (BLASTX) analysis, the EST-SSR-containing unigenes were divided into three classifications according to their functions, namely (a) a biological process; (b) a cellular component; and (c) a molecular function. The unigenes classified as being involved in a biological process were shown to have a putative function in the metabolic process, primary metabolism process, response to stimuli, biological regulation, cellular process, biosynthetic process, and so on (Figure 2a). The unigenes classified as cellular components are shown in Figure 2b. In the cellular component category, the greatest numbers of genes were found in the terms cellular component, cell, and cell part. The unigenes classified in the molecular function category were shown to have a putative function in binding, catalytic activity, DNA binding transcription factor activity, transporter activity, nucleic acid binding transcription factor activity, and so on (Figure 2c).

Among the patchouli active components, the sesquiterpenoids and flavonoids are the most abundant. However, although some of the genes involved in the terpenoid and flavonoid biosynthesis have been isolated and functionally characterized, the global terpenoid and flavonoid biosynthesis pathways in *P. cablin* remain yet to be fully characterized. The gene ontology (GO) analysis showed that the functions of the identified genes are involved various biological processes. A large number of catalysts and transferases were annotated, which suggests that our study may allow for the identification of novel genes involved in the sesquiterpenoids and flavonoids synthesis pathways.

### 2.3. Polymorphism and Cross-Species Transferability

In order to validate the EST-SSRs and test their transferability across related species of *P. cablin*, 192 primer pairs were designed randomly and 48 accessions were selected, including 10 from related species that are widely used in traditional Chinese medicine and 38 *P. cablin* accessions. Out of these 192 primer pairs, 45 SSR markers were polymorphic (23.4%) and with allele variations from two to four. Furthermore, only 12 SSR markers were found to be interspecifically amplifiable, and 10–40% cross-species transferability in 10 related species were revealed. The characteristics of 45 EST-derived SSRs for *P. cablin* are given in the Supplementary Materials.

## 3. Discussion

In this study, EST sequences were obtained and used for the development of a novel set of functional markers. Among all of the EST-SSRs developed, the trinucleotide motifs were predominant, corresponding to 80.8% of the EST-SSRs, which was 7.5 times higher than the amount of the second abundant motif (dinucleotide, 10.8%). This may simply be reflecting the fact that trinucleotide SSRs do not cause frame shifts when present in ESTs, because they are multiples of three (i.e., of the number of nucleotides in a codon). This is not the case for di-, tetra-, and penta-nucleotide motifs. The most common SSR lengths were 12, 15, 18 and 21, which were all multiples of three, supporting the idea that there is a positive selection pressure for the in-frame SSRs in genic sequences [26].

EST-SSRs are derived from the expressed portion of the genome, therefore, they can be assayed as gene-based functional markers for diversity assessment, gene mapping, and marker assisted selection. The gene ontology (GO) classifies genes according to their function in three categories (i.e., molecular function, biological process, and cellular component) [27]. In our study, most of the identified unigenes matched unique known proteins that are present in the public databases, implying that the transcriptome sequencing yielded a great number of unique genes in *P. cablin*. A large number of unigenes were assigned to a wide range of GO functions, which indicated that the transcriptome data represented a broad diversity of transcripts in *P. cablin*. Similar results were also reported in other species [28,29,30,31,32]. The unigenes related to a biological process, such as the response to a stimulus and a secondary metabolic process, should be paid more attention when studying the medicinal plants whose activities rely on the content of secondary functional components generated by the response against a stressor. By employing transcriptome sequencing and gene annotation, such a large number of transcriptome sequences will provide an excellent resource for gene isolation and gene expression profile analysis in *P. cablin*.

In China, cultivars of *P. cablin* in different regions have different morphological characters and essential oil constituents. It was reported that *P. cablin* in China could be classified into two chemotypes, the pogostone type and patchoulol type, whose essential oils contain extremely high amounts of pogostone and patchoulol, respectively [8,33]. The pogostone type patchouli plant is mainly cultivated in the areas of Guangzhou and Zhaoqing in Guangdong Province, and is called ‘Paixiang’ and ‘Zhaoxiang’, respectively, in Chinese, whereas the patchoulol type *P. cablin* is cultivated in the areas of Zhanjiang in Guangdong Province and Wanning in Hainan Province, and is called ‘Zhanxiang’ and ‘Nanxiang’, respectively. Traditionally, Paixiang is considered an authentic herb, according to the traditional Chinese medicine theory. Additionally, the patchoulol type is mainly used in the perfume industry because of its higher yield of essential oil compared with the pogostone type *P. cablin* [33]. Unfortunately, Paixiang, which is considered a genuine herbal material, is becoming rare, because its cultivation has been greatly decreased as a consequence of the expansion of the urban area of Guangzhou city. In order to explore the genetic features of the authentic cultivar and to accelerate the development of genetically improved cultivars, it is desirable to understand the inheritance pattern of complex quantitative traits like yield and adaptability. This can be facilitated by developing DNA-based markers, such as SSRs, and applying them to estimate the level of genetic diversity and to construct a framework linkage map and QTL mapping for tagging the target traits [24,34,35]. Molecular markers are widely distributed in various regions of the genome. By comparing the polymorphism of molecular markers randomly-distributed in the genome, the diversity of plants can be evaluated and used to cluster the species, and to understand their phylogeny and phylogenetic relationships. In this study, the cross-species transferability of EST-SSRs could help understand the evolution of microsatellites in a phylogenetic context, and EST-SSRs on a genic loci might play an important role in speciation or gene functionality diversification during the evolutionary process. The development of patchouli EST-SSR markers provides a powerful tool for the study of phylogenetic *Pogostemon* genus and Labiatae family. More importantly, the EST-SSR markers developed in this study are a good resource for molecular marker-assisted breeding. Consequently, these EST-SSR markers are useful tools to map the genes related to bioactive components. This study, for the first time, reports the development of high-throughput EST-SSR markers in *P. cablin*, and assesses their transferability across a wide spectrum of related species and genera. The patchouli EST-SSR markers that have been developed are valuable genetic resources for investigating species relationships and for comparative mapping in the Labiatae family. 

## 4. Materials and Methods

### 4.1. Plant Materials and DNA Isolation

A total of 38 patchouli accessions and one accession each of 10 related plants (*Mesona chinensis* Benth., *Prunella vulgaris* L, *Salvia bowleyana* Dunn, *Stachys geobombycis* C. Y. Wu, *Teucrium viscidum*, *Dysophylla aurl* Cularia [L.] Blume, *Mentha haplocalyx* Briq., *Mentha spicata* Linn., *Leonurus artemisia* [Laur.] S. Y. Hu F, and *Salvia plebeia* R. Br.) were used for the examination of polymorphism and for the transferability of patchouli EST-derived SSR-markers (Table 3). 

Genomic DNA was isolated from fresh, young leaf tissues of patchouli accessions using the Cetyltrimethylammonium bromide (CTAB) method [36]. The genomic DNA was purified using the GenElute genomic DNA miniprep kit (SIGMA Aldrich, St. Louis, MO, USA). The DNA quality and final concentration were estimated by agarose gel electrophoresis, using a known concentration of uncut λDNA as a standard.

### 4.2. Transcriptome Sequencing of P. cablin

For the transcriptome sequencing, the young leaf tissues of three patchouli cultivars were collected and used to build up RNA libraries. The samples were immersed in liquid nitrogen immediately after collection and then stored at −80 °C. The total RNA was isolated using the Trizol reagent (Invitrogen, Carlsbad, CA, USA), according to the manufacturer’s instructions. The quality of the total RNA was checked on a spectrophotometer, the NanoDrop 2000c (Thermo Scientific, Waltham, MA, USA), and a 2100 Bioanalyzer (Agilent, Santa Clara, CA, USA). The RNA samples were stored at −80 °C until further use. Poly(A) mRNA was isolated with oligo(dT) beads. The first-strand cDNA was synthesized using a random hexamer-primer and reverse transcriptase (Invitrogen, Carlsbad, CA, USA). The second-strand cDNA was synthesized using RNase H (Invitrogen, Carlsbad, CA, USA) and DNA polymerase I (New England BioLabs, Ipswich, MA, USA). Then, the cDNA libraries were prepared according to Illumina’s protocols (San Diego, CA, USA), and were subjected to the Illumina Genome Analyzer’s proprietary sequencing-by-synthesis method. The sequencing was carried out at the Beijing Genomics Institute (BGI, Shenzhen, China). In total, three single-end cDNA libraries were constructed for each patchouli cultivar. The raw sequencing dataset for the *P. cablin* was submitted to the NCBI/SRA database under accession number SRP150583.

### 4.3. De Novo Assembly and Mining for EST-SSRs

The raw sequencing reads were first filtered by removing the invalid reads, and then including the reads with an adaptor contamination, with ambiguous ‘N’ bases at a ratio greater than 5% and reads with a more than 50% base with a quality lower than 20 in one sequence. The short clean reads were assembled using Trinity software to construct contigs. These contigs were used for the further process of sequence clustering, with the software TGICL (TIGR Gene Indices clustering tools) to form unigenes [37]. The 54,546 ESTs were searched for microsatellites using MIcroSAtellite (MISA) scripts (http://pgrc.ipk-gatersleben.de/misa/). The criteria for the SSR search by the MISA were repeat stretches having a minimum of six repeat units for dinucleotide SSRs, and five repeat units in case of tri-, tetra-, penta-, and hexa-nucleotide SSRs. The identified EST-SSR sequences were deposited in the Genebank so as to obtain the accession numbers. The putative function of the developed functional markers-encoding unigenes was found using the BLASTX tool of NCBI, assuming an *e*-value < 1 × 10^−10^ as a significant homology.

### 4.4. Designing EST-SSR Primers

The primer sets were designed using Primer3 [38] and the Primer3 interface modules scripts by the MISA developers at http://pgrc.ipk-gatersleben.de/misa/primer3.html. The parameters used for primer design were (1) a primer length of 18–24 bp, with an optimum of 20 bp; (2) an annealing temperature of 50–60 °C, with an optimum of 55 °C; (3) a percentage GC in the range of 40–50; and (4) a product size in the range of 100–400 bp. 

### 4.5. Polymerase Chain Reaction (PCR) Amplification and Polymorphism Validation

A total of 38 wild patchouli accessions and one accession each of 10 related species were used for the examination of the polymorphism and transferability of the patchouli EST-derived SSR-markers. The PCR amplifications of the genomic DNA were performed in a 10 µL solution containing the following components: 1× PCR buffer (10 mM Tris-HCl, 50 mM KCl, 1.5 mM MgCl_2_), 0.2 mM of each dNTP, 0.2 µM of each locus-specific primer, 0.075 µM M13 (−21) primer labelled at the 5′ end with 6-carboxy-fluorescine (FAM) and hexachloro-6-carboxy-fluorescine (HEX), 0.25 U of Taq polymerase (Fermentas, Vilnius, Lithuania), and 20 ng of genomic DNA. The amplification was performed in PE9700 (Applied Biosystems, Waltham, MA, USA) or T-gradient (Biometra, Jena Germany) thermalcyclers, using a two-step PCR protocol, with an initial touchdown cycle of 94 °C for 5 min, followed by five cycles of 30 s at 94 °C, 30 s at 60 °C (which was lowered by 1 °C for each cycle), and 30 cycles at 55 °C, and ending with a 20 min extension step at 72 °C. The PCR products were detected by gel electrophoresis in a 2% agarose–1 × Tris-borate-EDTA (TBE) buffer. The sequence analysis was performed on an ABI 3730 DNA Sequence Analyzer.

## 5. Conclusions

Currently, the development of functional molecular markers such as EST-SSRs is becoming a valuable objective with regards to plants, especially in marker-assisted breeding programs. In this research article, a set of novel EST-SSRs developed from cDNA libraries were identified and characterized. A total of 45 polymorphic EST-SSR markers were ultimately screened and confirmed across 38 patchouli populations and, interestingly, showed a 10–40% cross-transferability, in 10 related species of Lamiaceae. There is no doubt that these novel EST-SSRs will be helpful for future research on genetic diversity, population structure, evolutionary processes, linkage map construction, and QTL mapping for *P. cablin*. In addition, as a codominant marker, EST-SSRs are abundant in the genome, and can be used as an ideal tool for maker-assisted breeding, constructing genetic maps, and for the cloning of key genes for *P. cablin*.

## Figures and Tables

**Table 1:** Summary of the in silico search for simple sequence repeats (SSRs) in *Pogostemon cablin*.

Parameters Used in Screening	Data Generated by MISA
Total number of sequences examined	54,546
Total number of identified SSRs	1219
Number of SSR-containing sequences	1144
Number of sequences containing more than 1 SSR	67
Number of SSRs present in compound formation	37
Total size of examined sequences (bp)	36,417,906

**Table 2:** The number of non-redundant SSRs in a set of 1219 patchouli expressed sequence tags (ESTs).

SSR Motif	Number of Repeats												Total
	5	6	7	8	9	10	11	12	13	14	15	>15	
A/T	-	-	-	-	-	47	14	6	2	5	1	2	77
C/G	-	-	-	-	-	5	2	1		1			9
AC/GT	-	10	1										11
AG/CT	-	83	24	10	1		2						120
AT/AT	-											1	1
AAC/GTT	20	6	5										31
AAG/CTT	116	58	9										183
AAT/ATT	7	4											11
ACC/GGT	114	35	14	1								1	165
ACG/CGT	17	6	2										25
ACT/AGT	5	2											7
AGC/CTG	66	20	16	3									105
AGG/CCT	105	36	10										151
ATC/ATG	95	21	5										121
CCG/CGG	127	46	12					1					186
AAAGAT/ATCTTT	2												2
AAAGGC/CCTTTG	2												2
AACAGC/CTGTTG		2											2
AACCCT/AGGGTT				1									1
AATCCC/ATTGGG		1		1									2
AATCTG/AGATTC	1												1
AATGGT/ACCATT	1												1
ACAGCC/CTGTGG					2								2
ACCTCC/AGGTGG	1												1
ACTCCG/AGTCGG			1										1
AGCCCT/AGGGCT	1												1
												Total	1219

**Table 3:** List of 38 patchouli accessions used in the analysis of genetic diversity.

Resource	No. of Accessions Sampled	Origin	Latitude (N)	Longitude (E)
*Pogostemon cablin* (Blanco) Benth cv. Hainangensis	3	Longdong, Guangzhou city	23°12′20″	113°22′36″
*Pogostemon cablin* (Blanco) Benth cv. Shipaiensis	3	Longdong, Guangzhou city	23°12′20″	113°22′36″
*Pogostemon cablin* (Blanco) Benth	3	Longdong, Guangzhou city	23°12′20″	113°22′36″
*Pogostemon cablin* (Blanco) Benth cv. Hainangensis	6	Didou Town, Zhaoqing City	23°33′58″	112°43′03″
*Pogostemon cablin* (Blanco) Benth cv. Hainangensis	4	Liantang Town, Zhaoqing City	22°57′04″	112°27′54″
*Pogostemon cablin* (Blanco) Benth cv. Gaoyaoensis	4	Liantang Town, Zhaoqing City	22°57′04″	112°27′54″
*Pogostemon cablin* (Blanco) Benth cv. Hainangensis	5	Yingli Town, Zhanjiang City	20°29′51″	109°58′10″
*Pogostemon cablin* (Blanco) Benth cv. Hainangensis	5	Yingli Town, Zhanjiang City	20°33′51″	110°04′03″
*Pogostemon cablin* (Blanco) Benth cv. Hainangensis	5	Tanshui Town, Yangjiang City	22°04′25″	111°30′19″

## Supplementary Materials

The supplementary materials are available online.

## Abbreviations

The following abbreviations are used in this manuscript:

EST	expressed sequence tag
SSR	simple sequence repeat
RAPD	random amplification of polymorphic DNA
ISSR	inter-simple sequence repeat
SRAP	sequence-related amplified polymorphism
QTL	quantitative trait loci
BLASTX	Basic Local Alignment Search Tool X
PCR	polymerase chain reaction
GO	gene ontology
