# Supplementary material for: Development and Characterization of High-Throughput EST-Based SSR Markers for Pogostemon cablin Using Transcriptome Sequencing
Source: Molecules. 2018 Aug 13;23(8):2014. doi: 10.3390/molecules23082014 (PMC6222658; doi:10.3390/molecules23082014)
Supplement: Supplementary file 1 [file molecules-23-02014-s001.zip › Supplementary File 2 The Sequence of 45 SSR-containing Unigenes.pdf]

>Unigene56788
CAGCAGCAGCAGCATATGATGCAGCAGCATATGATGCAGCAGATCCAAAGACAGAAAGAC
GCTATTTCACGTTTCCCCTCCAACATCGATGCTCATCTCCGCCCACAGCATCATATCTTT
CAGCAACGTCCTATTAACCCTAATCCCACTTCCCAACAACAAATTATCCCTAATCAGCTT
CATCCCAATCCCAATCCCAATCCCAATCCCAATCCCAATCTTAATTCTTCTGTAACACCA
ACCAACAATAACCCACCGCAGCAGCAGCACAAAGCCTCTCTCCAGCTGGCTTATCAGGAC
GCCTGGCGGGTCTGTCACCCCGACTTCAAGCGCCCCTTCTCTTCCCTCGAAGACGCTTGC
GAGAGGCTATTACCTTACCATGTGGTGGCGGACTATGAAGCAGAAGAAGATGACAAGATT
CTTGATTCAGCCACTACAGGCCAAGTCCTTTCTCGCTCTCAGCAGTGGGACAATAATATT
GCAGCTAAAGTTGCAGAATTCACAGCCACATTCGAGAAACAAGTCCTTGCTTTCAATATT
ATTTCCCGTAAAAGAGGACTTGGGGAGTTTCGTACTGAGGAAAAGTTGATGATGGAGCAA
TTGCTGCTACAAGAAGAGAAGCGATCACTGATTGAATTGAGAGCTGAATTGGATTCGAGG
CAGAAAGCTAGCAGAGAAACTCATGAAGCAAATATGCGTATGGCAGCCATGGCTCATGTG
GATCAAGCTCATGCAGAATCACAAACCCATGCTGAGATGATGGCACGAGCCCCCATAAGA
GCAAATGCACTTGGTTCTCGAGGTAGTAGTACAATACCTGAAATGGGTGAGCAGGAGCGT
GATTTCCAGCAGGATGACATGATAAATGGGTGGGGCGCCAATACACAGAAGGATGAGAAG
GAGCCATCTGAGGACTTTCTGAATGATGATGAAACAGAAAATGGAGATGCAGCATTGCAG
AGTGAGTGGCCTGGAGGAGGTGAGTTAGATCTGAATACGAGG

>Unigene586
ATGTATAACGGAATAGGGTTGCAGACCCCTAGGGGATCCGGAACGAATGGTTACATTCAG
TCCAATAAGTTCTTTGTGAAGCCGAAGACGAACAAAGTCATCACGGATTCGGCCAAGGGA
TTCGAATCGGGTCAAGGAACAGCCGGCATGACCCGGAAACCCAACCAAGACATTTTAGAC
CACGATCGCAAGCGTCAAATCCAGCTGAAGCTTCTTGTGTTGGAGGAGAAGCTGATTGAT
CAAGGATATACAGATGCCGAGATTGCTGAGAAGCTTGATGAGGCAAGGAAGGCGCTTGAG
GCGAAAGACAAGGAGAGTGGGGAAGGAGGAAATAATATATCAGACAAGGTTTCAGCGACT
CAGACCCATCAGATAGCTGCATTGAAGGAAAGAAAGATGGAAACACTCAAAGCTGCTCTT
GGAATAGAAACCGAGGCTGATAAAGAAAAGAAGCGTGGTGATGCTGAGGCCCCAGATTTT
GAAGAGAGTCTTGAGGAGGGTGAACTAGCAAGCATCCACAAAAAGGATGCTGGGAGGGAT
GATGTGCGAAAAAATTGCAAAGAAAACTGAATCTGATAGGGATGAAATCAAGCGTCGCAAT
AAGGATAAATTCAAAAGGAGCCCTGATGATTCTTCCGATTCAGATAAAATTGTTAGGCGT
GAGACTAAAGAAAAGCGCAAAAAAGACAGTAGAAGTACAGGTGCTAGTGATGATTCTGAT
GCAGATGTTAGGAAGAAACGGAAAATGTCGTCTAGTAAACACCGAAAAGGGCATTACAGT
AGCTCTTCTGACTCCGAATCTCATTCTGATTCAGATTCAGATTCAGATTCAGATTCAGAT
TCTGGTGGGAAAAAATCTAAGATGTCTCGTCGCAGACATGATTCTGATGACGACTACAGC
TCTGATGAGAGTCCTAAGAGTGGAAAGGAAAAGTTAAAGAAGCCTTCAAAGAACAAACAT
GTAGACTCTGATGATGACTGTTCCGGTAAAGGCCGTGTTTCCAATATTAGTAGGTCGAAG
GGAAAACGACATGATCCTAGTGAAGTCCATGTTCATAAGTCGAAGAGCAGACGTCCTGAG
TACAGTTCTGATGATGACGGTCGACGCCGTCATGATGATGACAGTTTTGATGAAGACCAT
GTTTCATATGATAAAAAGTTGAAAAGCAAACAACATGATTCAAACGAAGGTGATGTTCAC
AAGTCAAAGAGCAGACTACATGAGTATAGTTCTGATGACGACCATAGACGCCATCGTAAA
GGAAATCAAAATCAAGAAGGTAGAGAAGTTAACTTTGATGATAAATATAAAGCCAATTAT
GGCCTCAGATACAAGTCTCTGAGGGAGAATGATTCTACTAAGAGCAGGCGATATGATTCT
GATGATGAATCATTAGATAAAGGTGAAAAAGGCCGATCACAACAGAAAACTGCCAAAGCA

GAAGACCATTCTGGCGATAATAGTAGAAAGATCAGGGTTGACAAGCAGGATAAAGTAGCA
GCAGACGACCATCCCCGTGATAATAGTAGGAAAATCAGGGTTGATAGGAAGGATAAAGTT
GCAGCAGATGACCATCCCCGTGATGATAGGAATATCAAGGTTGACAGACATGATAAAGTT
GGTCGCAAGTATGGTCAAGATAGTGACTCTGAAGAAGCGACTAAAAAAAGGGAACAATAT
GATAAAAGTAAGGACAGACTGAGTGCTGGAGGTGGTAGACACGATGGTCATTATGAAAGA
GATCGGAATCGTGATTATGGTAAGGAGGATATTCACAAGGATTTGAATCGGGCTTCAGGG
GCCAGAGGCAGGGAGAAAGATTCTGCGCATGATTTTGATGATAGGGAGAATAAAAAGTTT
AAGGATGATGGAATGGACACATTCAAGAAATTGGAGCAATTATACAAGTCGAAGGGAGAT
GGAACTGGAGATAGAAGTGAAGACGGGTTGAGGAGTAAGCGGAAATTAGATGATGGACAT
CAGAATGAAGAACCAGAACGGAAGTCTAGGAAGCTGGGCTCATCGAAGGAAACTGGATAT
AAGAATGAGGGTGATGATGATCGAGGTGATCCAACCCGTAATAGGGATGCATCTCGACAT
GAAAATAGGAAAGATAATGAGAACCGTGAAGATTACGGACGAAATAGGAGAGAAAGAGGG

>Unigene49299
ATGGCAACTGGATGGATGAAGTCATTGCAGTGCAAGTCAAAAGCATTAGACGACGTCGTC
CACCACCACCACCACCACCACTCCTTATCCAACTCATCCAGCTGCAGAAACAGTTATCAG
AGTCTCAAAGATATTGTCGAAATTACCAAGAAGCCCCGCCCCAAGAAGCCTAAGCCACCC
AAACGACACCCATCGCCGCCGTTTAAACGGCCCGTTCCGAGGAAACCGGAGCCCGATTTC
GAATTCCAGCCCGCCGCCAGAACCCGACCCGTCTCCGCCCAATCCTTCTTCCCTTCTCTC
ACCGAGCTTCCGAAGGACCACCCGTCGCGGAATGTGGTGGAGATAATATTCCACACCAGC
TGGAGCGAGAAGAGCTTCCCGGGTCGGGTCGATATGGTGTTCAAGGTCCAGAACCTGACC
CGGACCCTGAACCGGTTCGAGGAGTATCGCGGTGTAGTTAAACACAGGGCCGGCGGCGCC
GCCGGGAGCGACGGCAGTGAGGACCACGTTCGCTGCATTGCGGATGGGAATGAGGTCATG
AGGTTTTACTGCCTAGGCGCCGCCGGCGCGTACGAGGCCGGGGCCGGCGCGTGGATGCTC
CAAGGGTGCAAAAGAGCCGCCGTGTGTACGTATTCCGGTAGTGGTGCGGCTCATGAGAAA
GCCGGTGGGGGAGAGGGAGGAGGGCCATGCTGGTTTGCCGGGTCATAGCGGGTCGGGTC
GGGAAGAAACTCGGGATTGACTCGTTGCTTGTAGAGCGAATCGGGTATGACTCAGTGAGT
GGTGAAAATGGGGAATTGCTAGTATTTGACTCGCGTGCATTGTTGCCCTGTTTTCTAATC
ATCTACAAATTG

>Unigene42
GAATCTGCTTCCACTGCTGTACAATTGTCTTCTGCTGCTGCTGCTGCTGCTGCATCAGAT
CCTGGTGCAAAAGTAAAATTCGACTTAAATGAAGGGTTTACTGTTGATGATGGGAAATTT
GGGGAGCCTGTCAGTTTAGTGGCCTCTGGTCTAACAGCTGTGATGACTAATAGTTTGCAG
TTCTCTGTAAATTCTACCTCTACTACCCATCCTGCTTCCATCACTGTAGCTGCTGCTGCC
AAAGGCCCCTTTCTTCCTCCTGAGGACCTATTGAAATACAAGGGTGAACTTGGATGGAAG
GGATCTGCTGCCACGAGTGCATTTCAACCTGCTGAGCCCCGAAAAGCTTTTGAAATGCCT
CTGGGTCCTAGTTGGTCTGCCCCTGATGCCTCTGCCAGTAAACATAACCGTATTTCATTG
GATATTGATCTGAATGTGCCTGATGAAGGAGTTGTTGAGGAAATGGTCTCACGAGATTCT
GCTTCAGCCATTTACTTGGCAACTCATTCATCAAGTACTTCTGCAGCACTGCTGAATGAG
CGGTCCAATTTTATGCCTTCTGCTGTCATGGATCTTGATTTGAACAGGCTAGATGAAACG
AATGACATTGGGCTCTACTACACCAGGAGCAATCAAAACGGAGTTGGACCAAATGATTTA
CATATTCGGAAGGATTTCGATCTCAATAGTGGGCCTGTAGTTGATGATGCTAGCATTGAC
CAAATTTCAGTTAAACAACTAGTCAACGGAGGTGTAACATCGCATCTTCCTCCTGCAGGC

ATTAGAATGAGCAGTGTAGGGTTGAGCAGTTTCTCATCTTGGAATCATCCAGGGAGTGCA
TACTCTACAATAACAATCCCATCAATGTTACCTGATCGAGCTGAGCAGCCCTTCCCAGTG
TTTCCACCTGGTGGATCCCAACGAATATATGACTCCGCGAATGTTACCCCATTTAATCCG
GATGTGTTCCGAGGATCAGTCTTGTCATCATCCCCTGCTGCTGTTCCTTTTGCATCCAGT
CCTTTCCAATTCCCCGTCGTTCCTCTCGGAACTAGTTACCCTCTCCCTGCAGCCACTTTT
CCTGTTGGAGCTACCTCATATTCAGACTCCTCACCTGGTGTAAGAATCTTTGCACCAAGA
GTGAATTCGAAGTACCTGGGTCCCATTGGCTCTTTGTCACCCCAATTTTAGAGGCCATAG
GTGGTCTTCCCGACATCAGCAAAAATGGTGGGTTGGATAGAAACA

>Unigene34396
GAGAAGGAAACGTCGAGCCGCAAACGTCGGGAATCCGACTCGCATCGCGATAGGGAAGCG
TCTAGAGACCACCACCACCACCACCACCGCTCCTCCAGGCACGACGACGAACGCCATAGA
TCTGATGAACACCACCGCCGCCGTTCCGATTCCAGAGCTGAAAGAGAGGGAAGTCGAGAT
CGGGATACGCGTCGCGAGCGCGAGAGATCGCAATCGGTTGAGAGAAAGAAGAGGAAGGAG
AGAGGTGATAGTGAGGAGAGGGGCGATAAGAGAGCTAGGGTTAATAATGGAAATGAGAAA
CGTAGATTTGAGGAT

>Unigene20980
GGATACCAATACCTAGTCAACCACTTCCTCGTCTTCCTCCTCCTTCCCATCATCATCATC
ATCATCATCGAAGCCCTCCGAACCACCCCCGAAGACCTCCTCCACCTCTACAACTCCATG
AAAAACTTCACCTTCATCCAACTCATCACTTCTTCCTTCCTCCTCATATATGTCTTAACA
ATATTCCTCCTCTCCAGACCCCGACCGGTCTACCTCGTCGACTACGCTCTCTTCAAACCG
CCGCGCAGCCTCCGTGTCTCCTTCGCCGGATTCATGGAGCACGCCAGGGTCGCCCTGTCC
AACTACCCGAAAAGCGTCCACTTCCAGATGAAGATTCTAGAACGTTCTGGATTGGGAGAA
GAAACCTGCCTCCCTCCGGCTATACATTACATCCCTCCGACACCAAACATGGAACTCGCT
AGAGAGGAAGCCGAGCTCGTGATTTTCTCTTGCATGGATTCTCTGTTTAAGAAAACTGGT
CTGAACCCTAAAGATGTCGATATATTGATCCTGAATTGTAGTCTCTTCTCTCCGACACCG
TCTCTGACGGCGATGGTGATCAATAAATACAAGATGAGAAGCAACATCAGGAGCTTCAAT
CTTTCCGGCATGGGTTGCAGCGCCAGTTTGATTTCCATTGATTTAGGCAGAGATCTTCTC
CAATACCACCCAAACTCTAACGCTGTTGTAATCAGCACAGAAATTCTCACCCCAAATTGC
TACCTCGGAAAGGAGAGAGCCATGCTCCTCCCCAACTGCCTCTTCCGAATGGGCGGCGCC
GCAATACTCCTCTCCAACCGCCGCGCCGATCGCCGCCGAGCCAAGTACCGCCTCGTCCAC
GTCGTCAGAACACACAAAGGAGCAGACGACAAATCATACAACTGCGTTTCCCAGGAAGAG
GACGACGAAGGAAACGTCGGCATCAAACTGAACATAGATCTGATGGCGATCGCCGGAGAA
GCTCTGAAATCGAACATCACCACCATCGGACCTCTCGTTCTTCCGGCGTCGGAGCAGCTC
CTCTTCGCCCTCTCTCTCATCGGAAGAAAGCTGATCAACTCCAAGTGGAAGCCCTACATT
CCGGACTTCAAACAAGCGTTCGAGCACTTCTGCATCCACGCCGGCGGC

>Unigene1115
GGTGCGTGTCTTAGTCTCAAAAACCAATGGCAGAACGGGTTGAGGCGGCGGAACAGCGGC
GGCGCCACTTGGCCGGAATTTTCGTATGGTAAAAATGTTGTTAAGCTGTGGGATAGCTTA
GGATTGAGTTTGGATTTTGATGAAGATCTGTTCAATTATGATTCGGAAACCGTGGTTTCT
ACCTGGGATAATCATCGCGGATTTTCCGGCGATGTTTGGAATGTTCCGGCGGCGGCGGCG
GCGGCGGCGCCTACGGTGGTGTTGTTGGCGGAGCGTAGTGGTAAAGGAGACGGCGTGATT

CTGGGTGGGTACGATACTAGGATGCGACGTCGTGTTCCAGCTCTGTACGCGGAGTGGAGC
TCTGCGGCGGAGAAGGCGGCGGTGCGAGGTGTGTCTACCGGCGGCGTTGGGAAGGTTTAC
GTCAGAGATGGCGTGAGTGGGGTGCTAACGGTGGGCGATGTGAGGAATGTTAGGCGGCCG
TTAGAAAACGTGCGAGAGTCTAACGGCGAGACGTGGTGGGACGCTGACGCCGTTATTGTG

>Unigene71071
GCGGAGGAGAAGAAAAAAGAAGAGCAAAAGGCTGAGGAAGATGTGCCGGAAATAGTTATG
AGGGTTTTTATGCACTGTGAGGGGTGTGCTAAAAAAGTCCGGCGGTGCCTCAAAGGGTTT
CAAGGAGTGGAGGAAGTGATAACTGATTGCAAAGGAAGTAGAGTGGTGGTTCGAGGTGAG
AAAGCAGATCCGGCGAAGGTGTTGGAGAGGGTTCAGAGGAAGAGCCACCGCCGAGTGGAG
CTCATCTCCCCACTTCCTGCTCAAAAAGAGGTGGTGGACGTGGTGCTGGTCAAACCTCAA
GAGGATAAACACGAGGGTGGTGGTGGTGGTGGTGTGATTACAATGGTGTTGGGAGTG
TTCATGCACTGCGAGGCGTGTGCTCAAGAGATTGAAAAGCGTATTCAAAGAATCAAAGGA
GTAGAAAGTGCAGAGGCTGATTTGAAGAGTTCACAAGTGAGGGTGAAAGGGGCGTTGGAG
CCCCAAAATCTGATCGATTTTGTGTACAAAAGAACCGGGAAACACGCCACAATCATTAAA
GTGGATCCCGACAACAAAAATGAGGAGAAACCCAAACCACAACCAGAGAAGAAAGAGGAG
GAACCAATCAAATCAGGACAAGAAAACGGAGAACCGGCGGCAGTGGAGGAGCCGAAGACG
GAGATAAAGAAGGCGGAGATGTACTATTACTACCCACATAACATCCAGTTGGATCATCAA
CGCTTCGTGCAAGACATCAATTCTTATTCTTATAGTTATCCTCCACAGATGTTCAGTGAT
GAGAATCCTAATGCATGCTGTGTTGTG

>Unigene49178
ATGGAGGATAAACAGTTGAATTTCAATCAACCGTTTTTATCAGTTAGACGACATGGACCA
ACTCCAACCTCACAAAGAAGTGAGAGAAGGAAAATCAACACTTCTTCCACTTCCATTCCT
AGGCTTCCTTCTTACAGATCAGAACTGAAATCTGGTCCCATCAGAGATCCGGGGGCCATT
CCGTTTTTATGGGAACAGTCCCCCGGACAACCTAAGGAAGAGACGAAACCAAACGATCGG
AGCTCTGATGAGCCTCCGATCGCCCCAAAGCTCCCTCCCGGGAGGTATCAGCAAGATTGT
GGTAAAATTGCTGTAGGCTGTAAGAACCAAGTTGTTGATCTGCCTCATAGTAATGGGGAC
GAGAGACGAGACGTTGATGTTCCCAGTGACTCGCGTGCTGATCCATGTCTCGATGAGAAT
GTCGAGAGATGTAAAGAGACGATAGACGAGGAGGAGAGATCTGATTCGGAGGATAGTGAT
GGAGCATATGTGGATGCACTGGATACTCTGTCAAGGGCTGAATCTTCTTTCTTGAACTGC
AGCATGAGTGGCTTAAGTGGTGTAGATGTGAAACCCCCTGGAAACTTTTCGACTGAACGA
CAAGCTCGCGAGTTCATGATGGATAGGTTCCTCCCAGCAGCGAAGGCGATGGCTTCAGAG
ACCCCTCAACATGCACCCAAGAAACAACCGTTTCTTCAAAAACAGCCACAGCAGCCAAAG
AAGACAATAGATCAATCTAAGCCTTCATTAAGATACGGGCCGAGCTTTGCAGACCGATAC
ACCTATCACCATGACAACAGAAAAGGAGAAGAAGAAGAAGAAGAAGATAGTGATGATGAG
TATGATCAACGTCAAAATGTGCCTGCAGTATGCGGTTTATTACCTCGTTTTTGCTTGAAG
AGTTCACTCTGCCTGTTAAACCCTGTTCCTGCAATGAGTGTTAGAACCCGAGTACCATCT
AAGAAAGTACAACATAGATCTTCACCAGCTGGTTCTTACAGTGAGATTGATCGAAGAATG
GTCAAAACCCAGACAGCTGAACTTGATCAAGAGAACGGGAAGCTGAGAAACAGGAGTGGT
CATGTAGCTAGCCTTAGCACATCAACGGCATCTCTGCCATTTCTTGAAGATGAAGAAGTC
CTTTCTGCTGCTGAAGAAACAACGAATGCTCGAATTCAAGGTCTTGGATCGCATGCAAAG
GGCTTCAAAACCTTTCAGGAGTTTTTGGATGATCAAGGAAGTCCAGGGGAGTCGAGCTTG
GGAGATCCTATAATCGAGAAAACTCTCTATATAGATACAGTACAGAATTTTGAAACCTCC

GATTTATCGTCTTGTAAGCCAAAGACAGAAGCCAGCGTTAAGGGTGTTCCTAGCTTAAGG
GAAGAAGACGAAGAAGAGATCATCACGAAAAGGATTTGTCAAACGCACGCAATAGACTCG
TCTCTACAAGATTTGAAACAGTCGACTGCAATGGATGTAGACAAGAATTTGCTGCTAAAT
GCTGACACACTGTGTGATTCCAATGTTTTATCCTCGACTGACAAGTCAACTGTAAAACGA
GTAAATCAAGGAATCAACCCAAGTAAGGAGAATACAGACGAAGTGACCGAACAAGGAGCA
ACAGAGAGTTTAGAAAAACAGTTCTCTATAGAAGTTACCATGGGAAAATCTTATCAGTAT
CACTCCGGACTTCTTGCTCCACCTCCTTTACCAAAATCTCCGTCTGATTCTTGGCTTTGG
CGAACATTGCCCACTGTTACCACCAAAAGCTCGTCTCTTCGTTCATACCGTGATGCAGCC
ACAACAAATCCCGAAAATCAACTGAAGAAGGCACCTACAAGTGATATGAAGTGGGAAAAT
ATTGTCAAAGCCTCAAAGGAAATGCTTACGACCATACCAGAAACT

>Unigene6405
ATGAAACGCCCTCTCGCTGCCGGCGACGCCCACTCCACCGTCTCCGAAACCGACGCCAAC
AAGAAGATCAAACAACAAGATACAGAAGAAATGTCGAAAGCAGCAGAAGCCGTTCTAATG
GACGAGAATCTGCTGTACGAGGTGCTGCGGCGGGTCGACGACGGGCGCACTCTAGCCAAG
GCGGCGTGCGTTAGCCGGCAGTGGAGGCGCACGGCGCACGACGAGCGGATCTGGGAGCTG
ATCTGCACGAGGCAGTACCACAGCAGCCCGATGCAACTACGCGCCGTTGTCACGAGGCTC
GGTGGATTCCGTCGCCTCTTCTCGAGCCACCTATGGCCGCTACTGAAGCCGTCGTCCTCA
GCGCCGCCTTCGGTTTCCACGTGGCATTCACTTCCTCCGTCGCCGCCGCCGCCGCCGCCG
CCAGCGAATACTAAGGCGCGGTGGGGAAAGGATGAGGTGAATCTTTCGCTTTCGTTGCTC
TCAATTAGGTACTTTGAGAAGATGAGTTTCAATAAGCGAAGCAAA

>Unigene37860
ATGGTGGAGATGGAAGGCGGTCCTTCCTCCAAACAGACGCCGCCGCCGCCGCCGCCGCCA
ATCACCGATATGTTCCAGAAGTTTGCTCTTGCTTTCAGGACCAAAACCTACGAGCTCTTC
ACCGAAGATGCCGCCGCCGACTCCGATGGTGACGTCACCCTTCTTCTCGACTCCGCCGAG
GAGTTCATCCCCGACCAGAAAGTTGTCGTTCTTAAACCCGACTCCATTTACGCCCGGGAT
AGTTCAGATGCGCAATTAATCGGAGCTTTGATCCCTTCTCTCTTTGCCACTCTCTCCTCT
TTTGAAGCTACCTACCTACAATTTCAAGCGGCCCATGTGCCGGAAGTTGACCCCACTGCC
CTCGAACAGGCCGATAAGTCGATCGTCTCAATTCTACAGAAACTGAATGAGATGAAGAAA
TTATACAGGGATTCGAAGAGAATGAGCTTAGGTTTAAATTGTGGTTTTGAGTTTCCTGCA
GCGTCGTTTTTGGAATTCCAAGTGCAGGAGAATCAGAGCAAGCTTAGGGTTTTGGAGACG
ATTGTGAATTCCTTGCAATCTCAAATTGATGTTAAGGATGACGAATCAAGGGATTTGCGG
AGAAAATTGGAGAAAATCCGAGCTGTAAATGCTGATTTAGCGCGGAAATTGGGCATCAAA
GAGGCAAATAAAGGTTTGGGGATTGAAGTCTTGCTGACAATTCGGGTTTTCGAAGCCATG
TTAGGCGATTCAGTCAAGTCACTGCGTTGCTTCGTGAAGCTGCTGATTGATTTGATGCAA
AGAGCAGGGTGGGATTTGGAGGAAGCAGCTAATTCTGTTTATTCAGGAGTTGATTACGCC
AAAAAGGGTCATTTCCGGTATGCATTTCTATCTTATGTCTGCTTGGGAATGTTTCAAAAT
TTTGATAAGCATGATTTTGGGTTAAGCAATTCTGAAATTATCTGCAATGGTAATGGGATT
AAACATGGTGAAAATGGTGAAATTGATAGTGACAATGATGGTTATCTGAGGCAGTTAGTA
GAGCATGTTGCGAGCAATCCTATGGAGATTCTAGCTAAGAATCCAAAGTGTGGGTTTTCC
CTTTTCTGTGGGAGGAAGTATGAAGAACTTATTCATCCCACAATGGAGTCATCTATTTTC
AGCAATTTAGATAGGAAGGAGACTGTGTTGGATTCTTGGAAGTCATTGACTGTGTTTTAT
GAGTCATTTGTTAGGATGGCAAGCTCAATATGGTTGCTTCATAAGCTGGCATATTCATTT

AACCCTGTGGTTGAGATTTTTCAAGTGGAGAGAGGGGTCGAATTTTCGTTGGTTTACATG
GAGGATGTTTTGGGTAGATGTGGTTTGCTCGGAAAAGCTAGACCAAGAGTTGGGTTCACA
GTTGTGCCTGGATTTAAAGTTGGAAGAACTGTTGTTCAATCACAGGTTTATCTCACTAAC
TTAAAACTTGTGGAG

>Unigene33864
ATGTCAAATTTGACTTGTGCATCTGGTGAAGCTAGTGGCTCTTCTGTCAACAGAAATGAT
AATAATGGTGGTATGAATATCTATCCAACTCCACTCAAGAAGAAAAGAAATCAACCAGGC
CATCCAGACCCTGGTGCTGAAGTGATAGCATTATCACCAAGATCCCTTTTGGCCACCAAC
AGATTTGTGTGTGAAGTGTGTAACAAAGGGTTTCAAAGAGACCAAAATCTCCAACTTCAC
AGAAGAGGCCACAATCTCCCATGGAAATTGAAGCAGAGAACAAGCAAAGAAGTGAGAAAG
AAAGTGTATATCTGCCCAGAAGTTAGCTGTGTTCATCATGATCCATCAAGGGCACTGGGA
GATCTCACTGGAATCAAGAAACACTTCTACAGAAAACATGGTGAAAAGAAGTGGAAGTGT
GAGAAATGCTCCAAGAAATATGCAGTTCAGTCTGATTGGAAAGCCCACTCTAAAATCTGT
GGAACAAGGGAATACAGATGCGACTGTGGAACTGTTTTTTCAAGGAGGGATAGTTTCATA
ACACACAGAGCCTTCTGTGATGCTCTAGCACAAGAGAATGCAAGATCAATCACCAGAAAC
AATATCCCTCTACAAAATTCATCAACAACATCTCTCCAATCCCAATTCAATAATGTTTCA
CAATTTCCAATAAAGAAAGAGCAGCAAAACTTCACTACTCCACCATGGCTAAGCTGCTCA
CCAATCATTGGACCACCCCCACCCCACCACATTGATCTCACCACCCCATCATCATTAATC
TTTCAAGATCATTTCCCTCACAACCATCAAGATCCAAACCCTAACCCTAGCTTCCACCAC
CCACCACCCCCATCACCCTACTTCTCAGCCACAGCACTGCTACAGAAGGCAGCCCAGATG
GGATCAAAATCCAGCAACACCACCCCACATTTCCCATCACACAGCGGTGGTGTGTTTATG
AGGGCCCACCAACACAACCAATCTCCTCACGTGTCTGCCTCAACAACTGATTTTGGTACC
TCACGTGATGATTTCATCCATGGCTTGTCTTCATATGGGAATAAAGCTGCTGCTGCTGCT
GCTGCTGCAGTGACAGGTGCAACCTTAGGTGGCAATAGCAGCACCACCCACCTTCCTTCT
CCCCCTCTCAATATCCATGAAATGATGATGATCAACAATGCTGGTGGATTGATTGAAGCT
GCCTCAGCCTTTGATGAAATTACATTTGCTGAAATGCTGAATAATAATTCAAAGAATGCA
AGTGGTGGTCAGTTTAGCAGCAGCACCACAAGTCATGAAGGAGGAGAAAACATGACAAGG
GATTTCTTGGGATTAAGGCCTTTGTCTCAAACTGAGATCCTAAGTATGAGTGCTTTTGAT
AACTGCATCAACGCTAATTCCTCTTGTAATCATAAT

>Unigene48244
ATGGACAGAAATAGAGATGGGAGAAGAGCAAGCATTGTTGGCTCTAATGGCTTCAACAGA
AGAAGACATCGGACGAACAGTCTCAGAGACTCACCAGATGAAGATGGCGGACAGGAGTTG
CAGGAATCCGTGAGGTTAAGAGATAGAGCGAAGAAAGATCGAGATCGAGACAGGGATAGA
GATAGAGAGAGGGAGAGAGACAGAGAGAGGGAAAGGGAGAGAGAGAGAGATTTGAGAGAG
AGGCCGAGCAGGAGTAAGAGAAGAAGAGGAGAGAGATTAACCAGAGATGATATTGGAGGA
GATGATACTTCAGAAGAGAGTGTCAACGATGAGGAGGATGAAGAAGACGAAGATAACCCC
TCCGCCTCCGCCGCCGCCGCCACCGCCACCGCCACCGCTGGGACAAGGTTGATGACGCCG
CCGGTTCACCACCACCACCATGGCCATCTCAGCAACCACCACCACAACCATAGCAGTAGT
TTTAGTCAACAACAACAACAGCAACACAATAGCAATAGTACTAGCAATATTGTAAGTAGT
AATCACCATCTTCAACATAGGAAAACCTTCCCTCCCTCTTCTGCAAAAGTCTTGCGAGCG
CCGCCGGTGTGGAAATCCGGCGACGAAATGATCGGCGTCTCCGTTCCAAGAAAAGCTCGT
TCAGCGTCTACAAAGAGGTCGCACGATTGGATTTCAAGCAGTAGTAACAACAACAGCGGC

GGTGGAGTTTCCGGCGAACAGAATCTCGGGCAAGCCACGTGTTCTCCGGCGAGACAAGGC
GGCGTTTCAACGGCGACGCCTTCTCCAGCTGCTCCTGTATCTCCATCCACTTCCAATGCT
TCAATGAAAAAGAAGCTCAAGCCGAGCGTCAACCACTCCGGGCCGAAGCTGAAGCCGCCG
AAGGTCTCATCGAAGCCGAGTTCTTCGAATCCGGAAGAACTGGAGATTGAAATTGCGGAG
GTGTTGTATGGATTGATGACTCAATCTCAAGCTCCATCGAAGAAAGAGGAATCGAGAGAA
ATCAATCGATTGAATAACGATGCCAAATCGCGTAATTCATCGCCGATTTCTAATTCTACT
TCGTCAAATAATCTCAATTTGGGACCCAATTCGAGTCCCCTTTCAGCTGTCGCTCCGAAG
AGAAAGAGGCCGAGGCAAGTGCTGGAGAATTCGAGCTTCGGCCACCGGAGTAGCTCCGTT
TCGGTGAAACCGGATGCAGATCAGGCGCCGAAGAGTGAGACTCAGTCTCACAACTTGGAT
AAAATCTCCGGATCCGTCGCTGAAAATGGTTACGGAATGGGGGTAATTCGGTCAACCTA
CAAGAACAACAGGCTGACCCACCGGCACCGACACCGGCACAGGCACCGACACTGACACCT
ACACCGCCGGAATCGATGAAATCGGATTCGGAATTGCATCCTGTAGCTGATGAATCGAAG
GAGAGAAAAGATTTGGTAGCGAAGGAAGAAGCGAGCTCACCCAAGGAGAAGGAATCCGCT
GTTGTTAGAGCAGAAGATAGCATCAATGACGATTCACCATCGACAGCGACCGCTTCATCG
ATTCCAACGACAATTAAAGCGAGCTCCATCGGTGCTGAGATTGAGAAACAGAAGGAAGAA
AAGTTCGAAATAGATCTGATGGCGCCGCCGCCTCAAGTTCGATCATCTCCGGAGAGGGAA
ACGAAGATTGATTTTAGGAATCCATCATCGCTGGATCAAAAGCCATCTTTACCGAATGTT
GATTCAAATTCATCCAAGGTCAAAAATCAGGAAGGTGAGAACTTGAAGAGTGCAAGTGGG
AAAGAGCATTCATTAAATGCAGAAAGGAAAGGTAGAGTTTCAGGAGAAGAAAGTGATTCA
CACAAAGCAATCGAGAAGAAACCGAGGAACGTTGATTTACATCTCGATTTGGAGAAGTGC
GACGGCGGCGATGCGGCCGGAAACACAAAGTCACAGTTGCAGAGTCAAAAGCAACATAAG
CAACAAATGCCATTTTCTGAGAAATCTGGCCATTCGACGAATTCATTGCCTTTGCCAATG
TCAATGGCTGGTTGGCCTGGTGGGCTTCCTCCCATGGGATACATGGCCCCGTTGCAGGGT
GTCGTTTCGATGGACGGAGGCTCCGTTGCACCGGGCCATATTCAGTCGTTGTTCTCACAG
CCTCGTCCGAAGAGGTGCGCTACGCATTGCCACATTGCTCGTAACATACACTACCTGCAG
CAGTTCATGAAGATGAACAACCCTTTCTGGCCCCCGCCTGCCAGTTCTGCCACCCTGTTC
GGGTCGAAACCTTGCAATCTGAACGCCGTGCCAGCCGGAGATATTAGAGGTGTTGGCAAT
TCGCAGGATAAGGGGCAGAGCGTTGCTGCAGTTTCTAATCACGGTGGGAAGGATAAAGGC
TCTCAATCGAACAATTCTAATCCTGATGCTGCTGCTCAGAGAAAGCAGCAGATTCTAATC
CAGCAAGCAATGCCTCCTAGTAATTTGCTTGGGCCTGCTTTTATCTTCCCTTTAAACCAG
CAACAGTCTGCTGTAGCACCTCGGCCTAGCGGTAATGCCAAGTCTCCGACTGTTGGGCCT
TCCCCGAACGCCCCCACTGCTGCGGTGACCAGCACTTCATCAGCAGCAACGATGAGCTTC
AACTATCCTAATATTTCGAATGAAACGCAGTATTTGGCGATCTTGCAGAACAATGCTTAC
CCGTTCCCCATGACGGCTGTAGGGGCGCCACCTAATTTCAGGGGCAATCCGGCTCAGGCA
ATGCCTTTGTTCAATGGCTCCTTCTATTCGTCCCAGATGATTCATCCGTCGCAGATTCAG
CACCATCAACCGCCGTCTGCTCAGTTGATGCAAGCTCACCCGAATGCAAGTTCGTCTAGT
GGATCCTCTTCGTCTCAGAAGCATTTGCAGGGCCAGAAAACTCAGAGTGGCGGCGGTGTT
TCCGGTGGTTCTGGAAGTGCAGGGTTGCAGAGCTTCTCGGCCCAGAAAGCTCAGCCACCT
CAGCAGTTATCCCATAATCAGTATGGGCATCCGCAGCGACCGAGGCATCTAGAGGGCGAG
CCGGGTAGTGAAGACGGTCCACCCTCGACTGACAGTCGAGGGTCTAGAGCCACGATGAAC
ATTTATGGTCAGAACTTTTCGATGCCCATCCACCCGCAGAATTTCGCGTTGATGGCCTCG
CCGGCTGCTCTGGCTGGCGCTGCTGCTGCTTCGGCTACTGCGACGAGTGCCAGTAGTAAT
CATCAAATTGACAAGAAAGCCCATCAGTCGCAGCAGCAGCAGCAGCAGCAGGGAGTGAAG
AATGGTTCAGAATCTTTGCCCCCACATGGATTTGCAATGTCCTTTGGTACCATCAATGGA

GCTACAACTGGCCAAGGGATGGATATGGCATCCATGGCTCAAAACCATGCTATATTCCAG
AGCTCCTCCGAAGCAGCCGCCCGGCAGAACATCCAGATGATGGCTGCAGCTCAGGCTGCA
CAGAAGAAGAGTTTCCGAATGTCTGATGATGGTAAATTGGGAGGCGTTGATTCGTCCACC
GGAGACGATGAAAGAAAAAGCATGGCTGGGAAGGCCCTAGGTGGTGGAGGTGGACAATCC
ATTGCTTTTACTAGGCAAGATTTGGGTGATGGATCTGTTGCTTCCATTCAGGCAAACAGT
GTGATTGAGAGCTCTGCTCGATCTCTAAACGTCGCATCTGGAGGCGCCCGGACCTCTCGA
TCCATGACAACGAATGCTGTGGGAGCTATCAGTAATGTTCAAGCTCAAATTCATCAGCAA
CAGCATCAGCAGATGCTGCAACTTAAACAGCATCAGCAGCATCAGCATCAGCAGCAGCAA
CAACTGGCGGCTGCAAATCGGAGCAAGGGGCCTGTGGCTAGCACCGGGAGTATGTACACC
GAACATCTGAATTCATCGTCTGCCATGGCTGCCAAGTTCCCAAACACACTCTCTGGCTTC
CCACAGAACCTTGTCCAGAACAACAGCAACAGCCCAAGTCAATCCCCGCAGTGGAAAAGC
TCGTCGCGAGCGCCTACGCCTCAAACACCGTCGTCTCTGGTCTCATCAACCACGACGACC
CTTAAAAGCCTTCAACAACAGCACACTCGAAGCCAACCACAAATGCATACTCAGATATCC
TTTGGAGGAAATCAGAAGCCAGCTACTGCCTCACAAGGGCAGGCGCCACCGAGTACCAAC
CAGACGCCGTCGTCCCCCATGATGGTTGGCTCTCCCACGACGTCTTCCATCTCCAAAGGA
GCAGGTGGGAGCCCAAGAACAACTTCATCAGCTTCCACGAACAACAAAATGAGCCAAGCT
TCGTCGTTGTCAGCACAGCCGCCGAAGAACTCATCCTCTGTTCCTAACCAGAAGTCTCCA
TCAATCCTTGGAAACCCTCACATGGCTTCTTCCTCCCCCAACAATGGACCGAAAGCTCAA
ATGCAGCAACAATCACAGCAGCAGCTCCCGAAAACAATGCAACAGGCACAGCTGTTCTTC
TCAAACCCATATGCCCAAAGCCAGCCTCCTCATCCTTCCACTACAAGTTCAACAACATCT
GGCCCGAGCGGATATTATATGCCACGAAGACGGCCGGAACAACACCAGCAGGGACTGGGT
GGTCCGGTGACATCCACCGGAGGGTTAACACTGTGCCCTATTGTTACACTGGGGAGCACA
AACACGAACGATCCAGCTACAGCAATTGCAGCAGCTACTGCTAATGTGAAGGGTGGTGGA
TTATCCTCACAGGGTATAATTCATGCCACTCAATTTGCTGCACAATCTGGTGGAACCATA
TTACCAGCTGGTTTCTCTTATATGCATCCCATTCCAGCTGCTGTTCAGGTGAAACCTGCA
GAGCAGAAACTACCTGCTGGAGATGACAATTTACATCCATGGCAGCCTGAGAAGAAG

>Unigene92831
ATGGATGAAGATAGCCTGAGGAATTGGGGTTACTATGAACCTCCCCTCAAGGGGCATCTC
GGTCTCCAGCTCATGTCTTCTCTGGCAGAGCGAGACACGAAACCTTTCCTAACCGGACGA
GACAATCCGGTTATGGTTTCAGCCAACGGGGCCTTCCATCCCAGGGATTGTGTGGTTACT
GAGCCACCAGTCACTCATATGGACTATGTAAGAGATAGTTGGATTAATCATAGGGAGAAG
TTCTTGCATATGTTCCCTGGAAACCCTTATAACCCTGTTATTCCCGAGTCGTCCGCCTCC
CGCCACCCTATCCAGATGTTGCAGCAGCAGCAGCAGCAGCAGCCGGATACGACCAAAGAG
ACCAGGCCTAGCATGGAAGAACCAGTTGTTAAGAAGGAGAACCCCCCTACCAAGAAGAGG
GGTGCTGCTACGACCCCAAAAACTCCAAAGGCGAAGAAGCCTAGGAAAGTAACCGAGCCA
AAGGAAAATGGTAGCAATCCATCAGTTCAACGTGCGAAAACAGCGAAAAAGAATGTGGAG
GTTGTTATAAATGGGATTGATATGGACATCACTGGGATCCCAATCCCTGTTTGTTCTTGC
ACCGGCACTCCACAGCAGTGTTACAGATGGGGCTGTGGGGGCTGGCAATCGGCATGCTGC
ACCACCACCATATCAATGTACCCATTGCCAATGAGCACGAAGAGACGTGGGGCTAGAATC
GCAGGTCGAAAGATGAGCCAGGGCGCGTTCAAGAAGGTGTTGGAAAAACTCGCCTCCGAA
GGGTATAACTTCGCTAACCCCATCGATCTGAAGACTTATTGGGCTAAGCATGGCACCAAC
AAGTTCGTGATAATCAGA

>Unigene37802
GAAGAGCTGGACTGTGTGGGTACTGGTTTGGGCGTTGAGTGCGCGGTGAGTCCTGCTGAT
GATTCGTCGATTAAACTTGAAGAAGAAGAAGAAGAAGAAGGTGAGAAATCGGAGATATGG
AGGTTGGATTTAGTTCAATTGGCAGGGTTTGCTTTAGAGTGGGGGGTTTTGATAGCGCCC
TTCTTCTTCTGGGGCACCGGCATGGTGGCGATGAAGCAGGTTCTACCCAAAACCGGCCCT
CTCTTCGTGGCGGCGTCTTGGCTGATTTTGGTGGGT

>Unigene49168
ATGGCCAGAAAACGAAAATCACGCGCCGCTCTGGAACCCGAAGTCGTACAGCAAGCTGAG
CCCGAGCCCGAGCCCGAGCCCAAACAAGAGCCTGAGCCAGAGCCTGAGCCTGAGCCCGAG
CCAGAACAACAATCCGTGAAAACCGAGCAAACCACTGAAGGAGGTGAGGAAGAAGAAGTG
GTTGAAGTCGAAGAGGAGGAAGAGGTGGGAGAGGGCGAGGAAGAGGGAGAGGGAGAAGAG
GAGCAGCCGGATACTATGGAGGAGGAGGAGGAGGAGGATGAGGAGGAAGACGAGGAGCAA
AAAGATGACGATGTTGCCACCGACGCAGAGAACGAGAAAGGGGTTGAAATCGCCGGGAAC
CCGTCTGAGAATGCTGTCTCAGAAAATGGCGGTGAAGCTGAAGGGCGGGGCGAGGAAGGG
GAAGATGATTTAGATGAAGAACCGATCGAGAAGCTCCTCGAACCATTTTCTAAAGACCAA
TTAACTCTTTTGATCAAAGCAGCTGTTGCTAAACACCCGGATCTAGTTGAGAACGTGCAC
AAATTGGCAGATGCCGACCCCGCACACTGCAAGATATTCGTGCACGGCCTCGGTTGGGAG
GCGAATACCGAGACAATTACCTCTGTGTTTGGGAAGTACGGAGAGATTGAGGATTGTAAG
GTGGTGAGAGACAAAAACTCTGGAAAATCTAAGGGTTATGGCTTTATTTTGTTCAAGCAC
CGTAATGGGGCACGTCGCGCTCTGAAGGAGCCTCAGAAGCTGATAGAAGGTCGGATGACT
TCATGCCAGCTGGCCTCAGCTGGGCCAGTTCAGTCGCCTGCCCCGACTGCAGCACCCGCT
GTTTCTGTACCACCTGCATCAGAGTACACACAGAGGAAGATTTATGTGAGCAATGTATCT
GCAGAACTCGATCCAAAGAAACTTTATGATTACTTCTCCAAATTTGGGGAGATTGAGGAA
GGGCCTTTAGGGTTGGATAAGCAGACAGGTAAACCTCGAGGTTTCTGTCTGTTCGTGTAC
AAGAGTCTCGAGAGCGCAAAGAAGGCACTGGAGGAGCCACACAAAAATTTTGAAGGTCAA
ATGCTGCACTGTCAGAAGGCAATAGATGGCCCGAAGCACAGTAAGGGTCACTTTAACCAG
CAGCATGGTCAGCAGCAACAGCAACCTCATCAACATCATCATCAGGGGCACCAAGGGTAT
TACCACCATCCGGCCAAGAAGGGGAGGTATTCTGGAAGTGGAGTAGGGCACACTGGTGGA
CATTTGATGGCTCCAAGTGCTGGACCAGCTGTTCCTTCAGTAGGGTATAATGCCCCTGCA
GCTTTAGGACAGGCTGTGGCAGCGTTGTTGGCCACTCAGGGGGCGGGTTTGGGTATTGGA
AACATACTTGGAGGGATTGGAGCAGGAGTGAATCCACAGGGAGTACCACCCATGATGAAC
AATGCAGGTTACGGAGGTCAGGGTGCTGGTGGTGGATATGGAGGTCAACCGGCAATGCAG
GGAGGTTATGCAGGCCAGCCGCAGATCAGTCAAGGTGGTGTCCGGCCACATCAGGGTGGG
GCCCCCTACATGGGCCATGGTCACTAGATTCTTCATAAATGCATCAAAACTTTTAGTTTT
GGACTGGTGATTCTTCTGAAGTTC

>Unigene33516
AAATTAAATTCAAAGCCAGCAATGGGTCAGTGTTACTCTGGACCATCAACACACAAGGAC
GATCAAGAATCTCCACCACCATCGTCGTCGTCGTCGTCGTCGAGGAGCTTGAAGAATAGA
TGTGCTGCGGTGGCGAAGGAGAAGCGATCAAGGCTTTACATCATGAGGAGATGCATCTTC
ATGCTCATCTGCTGGCACAAGTACGATGATTCC

>Unigene62496

AGCGACTCTTCTACTTCTACATTCAACATCGACGCCCTCGATCACGCCGAATTCGAGGTC
CAAGTTAACTCGGGGAAGAAAAAGCAGAGAGTGGAATATGTTTTGCCCGTTGGATTTCTT
GATCCAACCAGCCCCGTTTCGAGGGTTTCGGTGAAAGAAGAGAGGGTTGATGATGCCAAA
CGTTCAGAAAATTCGAAACAGGACACGATTACTACGTTCACTCTGCCGCCGAGTTGCAAA
CAGTTTTGGAAAGCCGGTGATTATGAGGGCGGCGGCGGCGGCGGCGACAAGTTTTCCGAT
GATGACATGGATCATCTGAGGGTTCATCCAAAATTCTTGCATTCAAATGCC

>Unigene56323
CCTCCCCAAAAAAAGTCAAAATGCCCACCACCACGACCGCCGCCGCCGCCGCCGCTACCT
ACACCACAATCCCAATCTCAGGCGTCGACGTCATTTCCCGATCCACGCAGAACCTATCCG
CCTGTCTCTCCCGCTGCCGTCCCTGGCCGGAGTTCTTCTCCACCACCTCCCTGCTCGATT
TCCCCTCCTCCT

>Unigene40899
CATCTCCGCCCCCGACCCTCCTCCCAACTCCGCCCCCTCAATTCCCATTCCTCCTCCTCC
TCCTCCACCCTCACTCTCCGCAACCCCCTCAACCTCTCTCTCTCCCCAAAAACCCACAGA
AGACTCATCGTCTCTGCCGCCGCCGCCGCCGACTCCGCCCCCGTCGAATCCCAACAGCCG
TCTCCTGCTCCTGCTCTACAAGGCGCCAAACCCATCCCCCTTATCATCTCAATTGCCGTC
GGCGTCGTCGTCCGCTTCTTCGTTCCGAAACCCCCCGAAGTCACGCCTCAGGCGTGGCAG
CTGCTCTCCATTTTCCTCTCCACCATCACCGGACTCGTGCTCAACCCCCTCCCCGTCGGT
GCCTGGGCCTTCATCGGCCTCACCACCTCCGTACTCACTAGAACACTCCCCTTCACCTCC
GCCTTCTCTGCCTTCACCAATGAAGTCATCTGGCTGATTGTTATTTCCTTTTTCTTTGCC
CGTGGGTTCGTGAAAACGGGCTTGGGGGATCGAATTGCCACCTATTTTGTGAAGTGGTTG
GGAAAGAGGACTCTCGGATTATCTTACGGTTTGGCTTTGAGTGAAGCCTTGATTGCGCCG
GCAATGCCTAGCACCACCGCGAGGGCGGGTGGTGTCTTCACTCCTATTATTAAATCCTTG
TCGTTGTCAGCTGGGAGTAAACCAGGGGAGCCCTCTAAGAAGAAGCTTGGATCGTATTTA
GTGCTTTCCCAGTTTCAGTGTGCTTGTAACTCTAGTGGACTTTTCCTAACTGCTGCAGCT
CAAAACCTGCTATGTCTCAAGCTAGCCGAGGGGCTTGGGGTGGTAATTTCAAACCCATGG
GTAACTTGGTTCAAGGTTGCAAGTTTACCTGCATTAGTCTCTCTTTTAGTAACACCGTTG
GTCTTGTACAAGCTTTATCCTCCTGAGTTAAAAAATACTCCTGATGCACCTGCAATGGCG
GAAAAGAAATTGGAGGCCATGGGTGGTGTGACAAAAAATGAATGGGTTATGATTGCTACC
ATGCTTCTTGCAGTTTCTTTGTGGATTTTTGGAGACGCTCTTGGCATTGCAAGTGTTGTT
GCCGCTATGCTCGGGCTATCAATACTCTTACTGCTGGGAGTGCTTGATTGGGATGACTGC
TTAAGTGAGAAATCAGCATGGGATACATTGGCATGGTTTGCAATTCTTGTGGGCATGGCT
GGCCAGTTGTCAGATCTTGGCATTGTAAATTGGATGGCTGGTTGTGTAGCCAAAACACTC
CAATCGTTGTCATTGAGCTGGCCGGCTGCTTTTGGCATTCTTCAAGCAGCTTACTTCTTT
ATCCACTACCTATTTGCTAGTCAAACAGGCCATGTCGGGGCATTGTTTTCTGCATTTCTT
GCCATGCATTTGGCATCCGGAGTTCCAGGTGTGCTAGCAGCATTGGCTCTCGCTTACAAT
ACTAACTTATTCGGATCTTTGACACATTATAGCAGTGGTCAGGCTGCTGTATATTATGGA
GCTGGTTATGTAGATCTTCCAGATATTTTCAAGTTCGGATTCCTCATCGCTGTCGTTAAT
GCAGTGATCTGGGGTGTTGTTGGGGGATTTTGGTGGAAGTTCTTGGGGCTGTAT

>Unigene33712
ATCAACGGTCCAGATCTCCTCCTCCGTTTCTGGCTCTCCGTTTATCTTCCCAACCTCCCC

CCTCTCTCCTTCCCCCCCAAACCCCCCACCCCCTCTCTCTCTCTCCTCTGCAACTCGATT
TTATATACATACTGATACGAGTAGAAGGAAGCTCTCTCTGAGGGGGTGTGTGTGTGAGAG
AGAGAGAGTAGAGAGATGATGTTGGGAAAGAGATCGCGTCCTCCGATGAAGAGAACTACG
AGCATGACGGAGTTCACCTTGGATCTCAACGGCGGCGCTTCTAGAACCGTCTACCAGCCG
TCGGATCCCCACAATCCTTTCAACGGCGGAGCGGTGGATCAACGGTACCTGGCCTCCGCC
TCCTCCGCCGCCTCTCCTCGTCCGCAGCGGCGCGCCTCGTCGGACTTCGTCGAAACTGCT
CATTTCCTCAGAGTCTGTTTCCTCTGCAAACGCCGTCTCATTCCAGGGCACGATATCTAC
ATGTACAGAGGTGACAGTGCTTTCTGCAGTCTAGAATGTAGACATCAGCAGATGACTCAA
GATGAGAGAAAAGAGAAGTGTTCCATGGCGGCGTCGAAGAAGGAAGTTGCCAGCGCCGCC
GCAGCCGGAACCAAAGTCTCCGCCGCCGGAGAGACGGTTTCCGCCGTG

>Unigene453
ATGAGTAAAGAAGAGTTTTTGAAGATCCAGACTTGTGTTCTTAAAGTCAATATTCACTGT
GATGGTTGTAAGCATAAAGTGAAGAAAATCTTGCAGAAGATTGAGGGTGTGTACACAACT
AAGATAGATTCAGAGCAAGGGAAGGTCACAGTTTCTGGGAATGTTGACTCAGCCACTCTG
ATAAAGAAGCTCATCAAGAATGGCAAACATGCTGAGGTGTGGGGTGCAGCTCCAAAGGGA
AACAACAATCACAATCAGTTCAAGAATCTCCAAATTGACAATGGCAAAGGTGGTGGTAAC
AAGGGGCAACAACAACCCCAGAAGGGGGGTGGTAATAACCAGCAGAAGGGGGGTGGGGGA
GGTCAGTGGCAGGGTCAGGGTCAGAATCCACATCAGCAACTTCAACAGATGAAAGGGTTT
CATCAAGATCTGAAAATGATCCCACCTCAGTTCATGAAGGACATGAAAATGCCCCCCAAT
GGCAACAATGCCAAGTCTGTGAAGTTCAAGTTGGAAGATGAAGATATGAGTGATGATGAG
CTTGATGATTATGATGACGACGATGATTATTTTGATGATGATGATGAGTTCGATGATGAT
TTGGATGATGCTCCTCTGAATAAGATGAAGGGCATGATGGGTAATGGCCATGGTGGTGGT
GGTGGAGGTCAGCAGATGATGGCCAAGATGATGATGCCAAATATGATGAATGGTCATCAG
CTTCAGGGGATGAAGCAGGGTGGGAATGGCGGCGGTGGTGGTGCCGGAAATGGGCAGAAG
GGCGGCGGAGGAAATGGTGGAGGCAATGCGCAGCAGAAAGGAGGAAATGGTGGTGGTAGC
AAACCTGTTCAGATGAATCATGGTGGTGGTGGTGGTGGGAACAACAATGGCAAGAAAGGT
GGAAATCAGAACCAAAATCAAGGTGGTGGAAAGGGTGGTAAGAACAATAACGGTGGACAG
AATGGTGGCGGAGGAAGTGGTGGTGGAGCTGGCAACGGCCACAATTTTAATGCAAATGGG
GCCAAGAAAGTAAGTGGAATGAATGATGGAGGTCATGGCATGCCTAACATGATGGGTATG
AATGGTGGTAGTGTGGGCCATATGGGGAACATGCCAATGGGGCAAATGAGTAATCTTGCA
GCCGTACAAGGCTTACCGGCAACCACCATGGGCGGTGGTGCCGGTGGTGGCGCAGGCTAC
TTCCCGGGCGGGGGTCCTGATCCCCACGTGCCCGGGAACCCCTACTATCAGCAGCAACTT
GCAGCTATGATGATGAATCAGCAACGAGCTCAAGGAAATGAGAGGTTTCAGCCGATGATG
TACGCCCGGCCACCACCGGCCGTTAATTACATGCCGCCTTATCCTCCATACCCGTACCCT
CCTCCACCCGGAGAACGAGCCGATCAGTATGCCATGTTTAGTGATGAAAACACCTCTAGT
TGTAATGTGATG

>Unigene48535
ATGCCAACCCTTTTGTTGAGAATTTTCTTTCTTTACAAGCTATTGAACGCAGTTCTACTG
TACTTGGTACCTAAGAAGTTGAGGACGTACCTCCCTGCTTCTTGGTACCCGTACACGCAG
CAGCAGCAGCAGCAACAACAACAACAACAACAACAGTATAACAAAAGAGAGCCCAATTCG
CAGCTTTCCTCCCCTTCTTTCTGCGCTCCTCGTAGAATGGATCCGACCGAGCTAAGACGG
GTTTTCCAAATGTTCGACAGGAACGGGGACGGGAGGATCACGCAGAAGGAGCTCAATGAC

TCCTTGGAGAAGATGGGAATCTTCATTCCGGAGAACGAGCTGACTCAGATGATCAATAAG
GTGGACGTCAATGGAGACGGCTGCGTAGACATAGACGAGTTCGGCACGTTGTATCAGACC
ATAATGGACGAGCGGGACGAGGAGGAAGACATAAGAGAGGCATTTAATGTGTTCGATCAG
AACGGGGACGGATTCATCACCGTTGACGAGCTGAAGACGGTGTTGGCGTCTCTGGGACTC
AAGCAAGGGAGAGCTGCGGAGGACTGCAAGAGGATGATCATGAGAGTCGATGCAGACGGC
GACGGAATGGTCAACTTCACCGAGTTTAAGCAGATGATGAGAGGAGGCGGCTTCGCCGCC
TTA

>Unigene20577
ATGGCTTCACCATCTGAACTAAGCTTTGATATCAAACCCCAAATGTATTCCATGCTTAAA
AAATCTTTGGGAGAGCAAACAACTGAGCAAACACAGAAGCTTGAAGAATTTCTAGCTCGT
TTAGAGGAGGAACGTCTCAAGATCGATGCGTTTAAACGTGAGCTCCCCCTTTGCATGCAA
CTCCTCACGGACGCAATGGAGGCGTCGAGGCAGCAACTACAGTCTGAGAGGGCGAGCCGA
TCTGAGGCGAAGCCAGTGCTTGAAGAATTCATTCCGCTCAAGAAGACGAGTGCATGTAAT
AATATGGAGATGAGGGAGGGGAATTTGTGTGGCGATAAGGCCAACTGGATGACATCTGCA
CAGCTTTGGAATCAAGAAATTGAAGGGACAAAAGCCCCTCAATCTCCAGTAATGGCATCT
TCTGATCAAGAAACTGATATCAAGTTAGGTTTGAATGGGAAACACAGAATGAAGGGGGGA
GGGGCTTTTCTACCCTTTCTGTCCAAAGATGGTGATGAGGCATTTCCAGAGTTAGCTCTT
AACTCATCACAAAATACAGAAGATCATATGGAAGATAAAAGAAATTCCTCAGAAACAATT
TCTAGAAGGGAGAATTCTGGTAAGGGTGGCAATTCTTGTGTTGAGAAAGAGCAAGTGAGT
GGGACATCAAATACAGCTCAAACTCATCGAAAGGCCAGGCGGTGTTGGTCGCCGGACTTG
CACCGCCGGTTTGTTAATGCTCTTCACATGTTGGGTGGTTCACAAGTGGCTACTCCCAAA
CAAATTAGAGAACTGATGAAGGTTGATGGATTGACCAATGATGAAGTTAAAAGCCATTTG
CAGAAATACAGACTTCACACAAGAAGGCCCAGCCTGAGCCCACAAGCGCCAGTCACGGCG
ACCCCCCAGGTGGTGGTCCTGGGCGGCATATGGGTCCCCCCAGAATACGCAGCAGCTGCC
GCCCACGGCGGAGCGCCAGCTGCTGCTGCGCTGTACGGCGCTCACGCCGCGGCCACTCAT
CACGTCTCGCCTTCTTTCTGCTCCCCCACACCACAGGTCCCTCAAGAGATCTACCCTGCA
ATAGCACCGCCACCTCCGGCGACCAACCACCAGCTACACCACCACACCATCAACGGCCAC
CACCACCACCACCAACAGCTGCATATGTACAATAAGTCTCCATTAACACAGAGAAACAGC
TCTCCTGATTCGGATATC

>Unigene11380
GAAGAAGACGACGATGAAGAAGCTCTCTCGCTCAGCGATCTCCCACTCATTCGTCGATCC
AACGTCGACGAAGAAGAAGAAGAAGAAATTACTCCACTCGTAGACTCTGCAACTCACGAT
CAGGATTTCGATTTCTGTTCTTTTTCGAAGGAATCGGAAATGTGCGCCGCCGATGAAGTG
TTCTTCCAAGGCCAGATTCTGCCGCTCCGCCATTCGATCAGCTCCGAAAAAGGATCGCTG
CTGTATTCCAGTCGATCCGTTTCCAGGTCCGAATCAATGGACCATCCCTATTCGGGTCGG
TTAATTTCCAGCCGGAGCAGCAGCATCAGCAGCCACCAATCGTCCAGCAGCGGCAGCTCC
TCCGCCACCGCCGCCGGGAATCACAATCACCGCCACAGACTACCGCCGCGCAACCAATTC
CATTCCCATTCCCACCCGAGCCCATCGCCGCGGCTCAATTTCACAACCCACAACCCGCGA
TTGAATCACCGGAAATCCGTGAGCAAATCGTCCGTCTGGAACATCCTCCGGCTCGGTCTG
GTGACGGCGCCGCCGGGAATGGCGGTTCACGATCTCAAAACGCGGAGCTCCGGGGGTAAT
TTCGGTAGCCGTCACAGCACGAGCAGCAATGGCAGCTCCGTTAGCAGTAGTGCGTCGAAG
AAGAAGAAGAAGGGGAGAGGGTTATTGGGTGGGTGCAAGTGCTCGATGGATGCAGTTGAT

ACAGTTCCTTCTAGAGTGGTGATAATCAAGAGGAGCGCCAGCGAGAGTGAGGTGGAATTG
CGTGAAACTGTAGGGGAGATAACCGGAGAGAAGATTGGCGCAAAGCAAACAGCGAAAAAG
TATCTGTCGCATCATCGAACATTTGAATGGTTGAAACAGCTT

>Unigene29777
CAACAACAGCAGCAGAAACCTGAGAGTACGGAGAATGACAATCGTTCTGAGTTCGAGAGG
GGCTTGGAGGAGTTAATGAATGGACATTATGATGAGTACATGTCCTTTGCTTCCTGTAGT
ACACCTAGAACAACTACTACGGAGGATGAGGAAGATGAAGGGGAACAGCTCATCAGGAGG
AGGAGGAGGTCCGATCTTGATGGTGATGACTTGGCGGAGTCTTCTGCTGCCAGGAGACGC
CACTCAAGGATTTTGAGCCGGTGGGCTGCCCGACAAGCGCAGGAGATGATCACCACAATG
GAGAGGAGGAATCGTGAGTCAGAATTAATGGCACTTGCTGGTTTGCATACTGTATCAACG
CTTGATTCGTCGTTCTTAAGGGAGTCTCAATCCCCTACCTCAAGGCGTGCTAATGTGGAG
AGGCTTGGGACTCGCGCCTCGTCCATCTTGCAAATGTGGCGGGAGTTAGAGGATGAACAT
GCACTTAATCGTGCTCGGGAGAGGGTGAGAGTGAGAAGGCTGCAGAGGCATAGTTCTAAT
ACTAGTGTATCCATGAATATGTCAGAGGGCAGAGAAACTGAGAATCATAGTAGCCTGGGG
GATGCCAGTGAGAGTGAGAATGATTATGCTTCTTGGTCTCACAATCAGTTGTCCCCACGG
AATAGGAATGGGGACAATGAAAATTCCAGTCGGGAGCAATCTCCTGATCTTGGAGAAATT
GAGAGAGAAAGAGTGAGGCATATTGTTCGTGGATGGATGGAGAGTGGCATCAGTGATAAT
TCTTCTAATGTTGTGCAGAGAACTGACAGTCCCAGAGCTGAATGGCTGGGGGAAACTGAG
CGTGAGAGGGTGAGGATAGTGAGGGAACTGATGCAGATGACAAGCCAGCAAAGAGGTTCT
CCTGATGGACACAGGGAAGAACATGATAATGGATCTGGATCTCAAGTTGCTAGTGTTCGT
GAAGGGTCTGTTGTTACCAGTCAGAATGTTCGCAGGGACAGGTTGCGGTTGCGAGGAAGA
CAAGCTTTACTTGATTTGCTTGTAAGAGCTGAAAGGGAAAGACAAGGAGAACTTCAGGGT
TTGTTGGAGCACCGTGCTGTTTCTGATTTTGCTCACCGCAATCGTATTCAATCACTACTT
AGAGGTAGATTCTTGAGAAATGAAAGGCCATCTGAGGAAGATAGATTACCTTCAATGGCA
GCGAGTGAATTAAATCAGTTGAGACAGCGACACACTGTCTCTGGCATGAGGGAAGGGTTC
CGCTTTAGGTTGGAAAATGTTGTGCGCAGCCAAGTAAATAGCCCTCCCGAAGCCTCATCT
AATAATAGCAACAGTGCTTCAAGACATCATCCACAAAATTCAAGTTCTGTGCAGGAAGTT
CAACATGAGAATGTGGAAACTGATAATCATCTATTAGCAGAGCGTGTCTGGAATTTGGAA
GGCAACACAGCTGTTCAAAATCTACATGTTTCTCCTGATCTAGGAAGTGATTGGCAGGGA
CTTGTTACTGAAGTTGAGGGAGAAAACCACCACCAGTTTTCTAGTGCGGATTCTAATGGA
TGGACCCATAGCCATTCAGAAAGCATAGATCTGAATTGGCAGGAAAATTCAATGTCTGGG
TGGCCTGCAGAAACTATTACCAATGAAAATAGAGGACAACAAATGCAAGAAGCTCAGGAG
GCTTGGCGTGATGTTGGCTCTGTAGAAGGTGTTGAAAATTGGACGGAAGCCCCTGCAGAC
CCTCCAAGAATGCTGCGGTTCATTCCATCTAGAAGGGTCAGCAGGTTCCACCCACCTGAT
GATGACAACGTGTACAGCATGGAACTTAGGGAACTTCTAAGCAGGAGAAGTGTCTCGAAT
CTTCTTCGCAGTGGTTTCCGTGAGAGCTTGGATCATTTGATTCAGTCATATGTAGAGAGG
CAGGGTAGATCTCCAATTGATTGGGATCTGCATAGGAACTTGCCTCTTGCACCATCGGCT
GAGAGGAATTCAGACATACAGAATGATGAGCGGAATGAGGATCAGGGTAATGCTATTGGT
AGACCTCCACATATATTGTCAACTCCTCCAGCACCACCACCACCACCACCCCTTTTGCAT
CAGGATTTGCATCGTTCTAGTTGGTCTCGTCACAATGTCCATCGTTCGGAGCTCGACTGG
GAGATGATCAATGATCTGAGAACAGATGTGGCAAAACTTCAGCAAGGTATG

>Unigene30364

CAGCAATCCGCCAAACAGGGGTACCCAAAGTCGAAGAACCAGAGTTTGACACCGAGTGAT
GATGATGACCCTGGATTGACAGCTGAGAATAGTGGTGAGGATGGGAAGAGGAAAACGTCT
CCATGGCAGAGGATGAAGTGGACGGACAACATGGTGAGGTTGTTGATCATGGTGGTGTAC
TACATTGGTGATGAGGTGGTTTCGGAGGGTAATGATTCTGTGTGTAAGAAGAAGGGTGGT
GGGGTGTTGCAGAAGAAGGGGAAATGGAAATCTGTGTCGAGGGCAATGATGGAAAGGGGG
TTTTATGTGTCTCCACAGCAGTGTGAGGATAAGTTCAATGATTTGAACAAGAGGTATAAG
AGGGTTAATGACATTCTTGGTAAGGGTACTTCTTGTAAAGTGGTGGAGAATCAGAGCTTG
CTTGATTCCATGGATCATATATCGCCTAAGATTAAGGAGGAAGTGAAGAAACTGTTGAAT
TCTAAGCACTTGTTTTTTAGGGAGATGTGTGCTTATCATAACAGCTGTGGTCATGGCAAT
GCTGGTAGTGGTGGTGGTGGTGGTGGCGGCATTCGTCCCTCTTCTCCGGAAGCTTCCACT
GAGCAGCCACCAGCTCAGCCGCAGCAACAGAGGTGCTTGCACTCATCGGAGAAGCCCCCG
GTGATGCCCAATTTGAACCGAGGGGAGAACGAGGGGTCTAAATTGGGGAAATTGACAAGT
GATGATGACGATGAGGATGACGAGGATGAAGACGACGAAGATGAGGATGATGATGATGAG
GAGGATGTTAATGATTCGAGGAAGAGGATGAGGAAAGGGACGAATCATTCGCAGTTGCTG
GTGCAATTGGACGCGGAGATGATGAACGTGTTGAGAGACGGGTCGAAGAGCTGGTCGGAG
AAGAGGCAGTGGATGAAGGCGAAGCTGATGGTGCTGGAGGAGCAGAGGGTGGGGTTCCAA
ACGCAGTCGTTTGAGCTGGAGAAGCAGAGGCTCAAGTGGCTCAAGTTTAGCAGCAAGAAG
GAGAGAGAAATGGAGAGAGAGAAG

>Unigene22730
GGAGGATGCTGTATAGCGAGGTATAAAGGTGGAGGGGCATACGATGTATCTAAGATGGAC
AGGATCCTGCTGAGGTTTCGTCCCATAGCTCCCAAACCAGCTTCTGTTAGCAGATCAGCT
TCCGGCAGCGACTCCACCGCGCCGGAGAACACCACCGCCCCACTCAAACGAGGTTCTGGG
AGAAGACGCCGTGTCGGTGTCGCTAAAGGTGGTAGTAAAGTTAGTGTTGGCAGGAGAAAC
AACAATAGGAAGAAGAAGAAGAAGAAGATATCGTCGGAAAAAGCCGACTCCGGTGGATCG
GTGACCGGGGGTGTCAATAACATCAAGACTTTGACGTTGCTTCCGGCGACACCTGTCGTG
AAAGAAGCTCCGATGCTCAGCTTTGAAGAAAATGGTGGAAATTATGATGGTGAGGTGGAT
CCGGCGACGGTGAGAATGAAGGTTGAATGCGTGACGGAGGCGTGGTTGGTCGACGGTTAT
CCGTACTGGATGGGGCGTACGGATGAGAAAATGGTTAGGGATTTGGAAGCTGACACGTGT
CCAGGATTTATATCTGATGGGTTGAATAGGGTGATGTGGACGAACGCAGCGTATCGGCGA
ATGGTATCGGGAAAAGGGGGGGAGAAGGTGGTGGTGGAGTCAGGGGTGGCGCTGCCGGTA
GGGAGTGCGGCGTTCACGTGCAGGGTAAGGGTGGTCACGTGCGGTAAAGAGAAGATGTGT
AGAACGGTGCCATGTGATGTGTGGAGGATGGAGTGTGGAGGGATTGCATGGAGGCTTGAT
ACCACGGCTGCTCTCTCCCTT

>Unigene12886
ATGGCAGCTCCAAATATGGCCACTATCACAGCATCTTTAGAGAGATCTCTGCAGAACTTC
TCGTTGAACCACCAACACGGCGGCAGCGGCAGTAGCAGCAGCAGCAGCAGCGGAGGGGGG
GCGGCGCGTGGGTCGGAATCGCCGCCGGTGGACGCGGTGGAACTCAATTCGCAATCCTCC
CTCCCCTTCCATTGGGAGCAATGCCTCGATTTAAAGAGTGGTGAGATATACTACATAAAT
TGGAGAACGGGGATGAAAGCGACGGAGGATCCGCGGACGACGGCGGCAGCGGAGTACATC
GGCGAATGTTACTGGGAGGAGGAAGACAGCAGCTCGTACGACAGCGAAGAGTCGTCCTCC
GAAACGTCGCCGTCTTCGTCGAGAGAGCAATGTAATGGTAACAATTATTATTATAATCAA
GAAAATGAGATTAATAATAATTCAAATAATAATTCAAATAATAATGTGCTGGTGGTGGCT

GGTTGCAAGAGCTGCTTAATGTATTACATGGTTCCCAAACACCTCCAAATTTGCCCCAAA
TGCTGTGGTCAACTCCTCCATTTTGATCGATCCGATAACGCCTCTTCT

>Unigene40408
AGAATCAACGGCGAATTCCATATTCCCACGTATGTGATTCAACACATACAGTCACATCTG
ACCCCAAAAGAAGCCGCTCAAACTAACCTACTCTCTAAATCATGGCACACCGCCTGGTCC
ACTGGCCCAATTCTCTATTTCGACTGCGCAAATTACCCTAATAAAGATCAATTTTGGGCA
TTCACGACGAAGACGATGCAGAGGTATGAAAATTTTAACTTAAAGGTGCAGAGTTTTACT
CTGATTATGAATAATAGGTCTGATTATAGTCGTATTAGCTGTTTAACTAATAATGAATGG
GATGCAAAAGACATGAAAATTGCTGTGGAATTAATATCGAAAGCGATGAAGATGGGCACC
GTAGATTTCAATTTTGGATTTTCTGATGCATGGAATACAAGTAAAAAATTCACTTTACCA
CATGAGGTGCTCGAATTCGAAACCCTAATCAGATTGTCTTTGTCTGGGTGCACAATTGAA
TTGGGAGGAGGAGGAGGAGGAAAGGTCAATTGTTTGGGATTTAAATCTCTTCGTTTAAAG
GATGTTCGGATAAAAAATAGT

>Unigene406
ATGGGTATATTTGAGGAAATGGGTTTCTGTGGTGATGTTGATTTTCTGTCAAGTGGTTGT
GGGGAAGTGGAGGTTTCACCAGTAGTTGAACCCACGATTCAGATTGATGAAGACTACAGT
GATGATGAGGTGGATGTTGATGAGCTGGAAAGGAGGATGTGGAGGGATAGATTGCTCTTG
AAACGGCTCAAGGAGCAGAAGAAGAGCAAGGAAGGGGGTGTTGATGATGTCAAGAAGCAT
CGTCATTCGCAAGAGCAAGCGAGGAGGAAGAAAATGTCCCGTGCACAAGATGGCATCTTG
AAGTACATGTTGAAGATGATGGAAGTGTGCAAAGCTCAGGGCTTCGTCTATGGCATCATC
CCTGAGAAAGGGAAGCCGGTGAGCGGAGCATCCGATAATCTCCGGGAGTGGTGGAAGGAT
AAGGTCCGTTTCGATAGAAATGGACCGGCTGCTATAGCCAAGTACCAGGCCGATAACTCC
CTCCCGGGGAAGAACGAGGGGTGTAACCCGGTCGGGCCGACCCCTCACACCTTGCAGGAG
CTCCAAGACACTACTCTTGGATCCCTGCTGTCTGCACTGATGCAGCACTGTGATCCGCCA
CAGAGGCGGTTCCCCCTGGAGAAGGGGGTCCCGCCTCCGTGGTGGCCCACCGGGAAGGAG
GAATGGTGGCATCAGTTGGGGTTACACAAGGATCAGGGGCATCCACCTTACAAGAAGCCT
CATGATCTGAAGAAGGCTTGGAAGGTGGGTGTTCTTACAGCAGTGATCAAGCACATGTCT
CCTGACATTGCCAAGATTCGGAAGCTGGTTAGACAATCCAAGTGTTTGCAGGACAAGATG
ACTGCCAAGGAGAGTGCAACATGGCTGGCTATCATCAATCAGGAGGAGGCCTTAGCTCGG
GAGCTTTACCCCGATCGCTGCCCGCCTCTGTCCTCGTCCGGTGGCAGCGCCACCTTCGCG
ATGAACGACAGCAGTGAGTACGATGTAGACGTGAACGATCACGAGGAGTCGAATTTCGAT
GTGCAAGAGCAGAAACCAGCCACACTTGGTCTGTTGAACATTGGTGGGATCAAGGATGAG
GTGATCACCAACTTGGATTTCTCTAGGAAGAGAAAAGGGGGCAATGAAGTGAACTTGATG
ATGGATCACAATAAGATATACACCTGCGAGTATCTTCAATGCCCTCACAGTGATCTTCGA
CATGGCTTTCAAGATCGAGCTTCAAGGGACAACCATCAGATGGCTTGCCCTTACACTCAT
TCCTCCCAATTCGCCGTCTCAAACTTCACCATCAACGACATCAAGCCGATCGTCTTCCCT
CAGTCGTTTATCCAGCCGAAGCCGGCTAGCCACACACCAGCAGAACCACCACCACAACCC
TTTGATTTATCAGGCCTTGGAGTACCAGAGGAAGGTCAAAGGATGATCAATGAGCTCATG
TCCTGTTATGACAACAACATCCTAGGAAACAAGAACACCACCAAGGAAGGCTACATTCAC
AGCCAAGGAATGGTGTTGGATGGCAACATGTTCGATGACGACAACAACAACAACAACAGT
AGCCACACACACAACGGTTCCATGTTCCGTTCGACGACGGATCGGTTCGACCAGTGCAAG

GTCATAAACACACCATTCAATCACAACACAAGTGACAATTTCCAGCTAATGTTCAGTTCT
CCATTCAACATCTCTCCTGTTGATTACACGGAGAATTTCCATGGAGGTGCAAGGGATAAC
CTGCAAAAGCAGGATGTCTCAATCTGGTAC

>Unigene56776
ATGGCGACCTACTTTCATGGGAATTCTGAAATCCAAGGCGGTGGCGACGGTCTTCAGACA
TTAATTCTCATGAATCCAGCCTACGTTGGATACTCCGAAACACAACCGCCCACAACCGGC
AACTTTGTGTTCCTCAACTCCAACTCCTCCGGGAACACCATCCACCACGCGCCGCCGTCT
CAGACCCAGCATTTCGTCGGCGTCCCCCTTCACGGCACCACTTCCACCACCACCACCACC
ACCTCCGCACAGGACCAGCAGCAGCAAGATGTTTCCGTCCTCCACGGCTTCCACCCGCGC
GTCTCCTACAATCTCTACAATCTCCCCATGGACCTCGGACCGGCGCGTGACATAACGCGC
GCGCAGCAGGGGCTGTCCCTGAGCCTCTCCTCCCAGCAACCACCGGGATACGGTTCCTTG
ACGCAGCCTCTGGGGTCGCCCCCGCGC

>Unigene10960
ATGGATCATTACAAGGTTTTGGGGGTTCAAAAGAACGCGAGCAAGGAAGAAATAAAGCGA
GCTTTTCGAAAATTGGCGATGGAATTTCACCCTGACAAGCATTCCCACTCCTCCAACAAT
TTGAGGAATTCCGCCACCCAAAAGTTCAAGCAGCTATCGGAGGCCTACGAGGTTCTGATG
GACGATCACAAACGCGCCGATTATAATCTCAAACGAAATGCGTATGGGACTCATCAAAGT
AGACGATATGATCACCGGTACGGCGGCGGCGGCGGCGGTTATGGTCATAGCTATAGCTAT
AGCAATACTAGATCTTCTGGGTATGGATTTAGCGGTGCAAGGACCAGTAGCTGCTCCGAT
GGTTTTGCCAAACTGGAGATGTTTATGCACTTTCTTAAGAAGAGGAGATTTGCGATCAAT
GCCTCTTTATTGGCATTACCTTGTATTATGCTAGGTGGGGCATTTATTGTTGATTCCTGT
GGCGAGGCATTATGGAAAATGCGTAATACCGGGAAATCTTTCGAGGAGGCAATGGATTCT
ATAGAGAGAGCTAAAGCAGACAAGGATAGTTCT

>Unigene52412
ATGAATTTCTTCAAATCAATTATATTAGACGATCCGGATCCCCCAAAACCCGAAAGTCCG
ACTGATTCTGGATCAAATCCCACCGTGAAACCATCTCAAGAAGATCACAGTCCAAACGAC
GTCGTTGAGGATGGTGGTGGGTGGAGTTTCGGAGGCTTGATCAAAACTTTAGCTACCAGA
TCCGAATCAGTGATCGAAACTTACAGCAGAGATCTGAAGGAATTTGGATCGGGACTCAGG
AAAGAGAGCGAGATCATCAAAGAAGCGGCTAGCCGAGCGGTGAAGGAACTTCCGGCGTCG
CTTGAAGCCGGAACTTCAGCTGCGCACGGTGTATTGGACGGAGTTCTGAAATCAACGGCT
GAGATCATTGCGAAGGAAACGCAAGCTTTTGGGTCGGATGGGGAATCGGAGACCCCGGAA
ACGAACCGAAGCATGAATTCGGGTCGGTATAGTCGCTTTGATGCGCAGTTGAGCGGTATA
CAGAGTGATTTGAATACTTTCTGTGAAGAGCCGGAGGATGTGGAAGAGTATAAGGAGTGG
AAATCAGGCTTTAAGTTGGATGATAAGATAGATGAAATTGATGAGCTGATTGGGGAGAAT
GGGAGCTTAGAGAGTGTTTATAAAAAGCTTGTTCCAGATTCGGTTGATCACGAGACGTTT
TGGGTTCGATATTTTTACAGAGTGAATAAGCTCAAGCTGAAGGAGAAGGTACGAGCGAAT
CTTGTGAAGAGGGCGATCGCAGCTGATGATGATGATGATGAATTGTCATGGGACGTTGAT
GATGATGATGGGGATGAGAAGAAGGATGCTACTGTGAGTGAAATAAAGACTCTTGAGAAG
GTGGAAGGAGGAGATGGAGCTGAAGGTAATGGAAGTTTAAATCTTGTTGAGAAAGGAGAG
AATTTGAATGATGGACCACAACCTACAACGGAGAAGTCCAATGTTCAGGTGAATGATGGA
GTTAACAACAATCTGGTCCTGTCGAAAGAGGAGAATCGCGATGGGGATAGCACATTGGAT

GTAAAAGAAGGGAAGTCTAATGAAGTAGCTGAAGTGAAAAGTGAGGAGAGGATGAAACCT
GAAGAAAGTGTCCAAGAAAAGGATAGCAAAGATAAGGGTGAGGCCGTGGAAAAGGGTCCG
ACTAATCATCGGGCAGAGACAGAGGAAGATGATATGGGATGGGATGAGATTGAGGACGTT
GATAGCGACAACGAGAAGAAAATCTCAACTAGCAGTCATGGAGAGAGACCAAACAGAGCA
GAGGTGAGGAAGCAGTTGATTGTTGCAGATGATGATGAGGATTTGAGTTGGGATATTGAA
GACGATGATGAGCCAGTGAAATCT

>Unigene20154
ATGGGGCCTAATTTGATGGATGAATTGGAGTTGTTCGACCAAATTGATGATTTGTTAGAC
TTCCCCCTTGATGATGAGTGTGGTGATGCCACCTTAGTTGATTCCACTGATTGCAAGGAT
TTCCCCTCTTGGGATGACGCCCTGCCGGACTCCCACCCTCTCTTCTCCGCCGCTCACGGC
GACCTCTCCGCCGAGCTCTCTGTTCCGTACGAAGATATCGTGCAGCTAGAATGGCTTTCG
ACGTTCGTGGAGGAGTCATTTTCGAGCGAAGGGTTGACCCTGGGGAAGGAGAACCTGCGT
GGGAGCATCACCTCCACCAACCACCATTTCCAAACAATGAGTCCTGTTTCTGTGTTGGAG
AGCAGCAGCAGCAGCAGCTCCTCTTCCGGAGGAAAGACGATGCCGCTTAGCCCCGGTCAC
CGTGGACCGCAGCGTGCAAGGAGCAAGCGCCCTCGACCTACAACGTTCAACCCTAGGCCG
GCAATTCACCTCGTCTCCCCTTCCTCCTCCTCTGGCGACGCCCCTGCCTATCCCCTGATC
ACCCCCGGGTTTTATTCGGAGTCTGATAGCTTTGCTGAGTCCTCACCTTTGAAAACAAAG
CCCAAGAAGATCAAAATAACCCTGTCCGAAGCTAAGCACCACGACCCTCCCGTCCAAGCT
GCTGTAAGGAAATGCCTACATTGCGAGATAACAAAGACTCCACAATGGAGAGCTGGACCT
ATGGGACCTAAAACATTGTGCAATGCTTGTGGTGTTCGTTACAAGTCTGGCCGGTTGTTC
CCTGAGTACCGTCCAGCTGCTAGTCCCACCTTCGTTGCATCCGTCCACTCAAACTCACAC
AAGAAGGTCCTGGAGATGAGAACCAAGATTGAGCCTAATACCACAGCT

>Unigene33886
TCGATCAATGATTCATCTTCAAGCTCGTCCAAGTTCTGGATTTTTCACGGCATCGCCGCC
GCCGCCGCCGCGGCAGCAGTCGGTGCCACTGTCTACTTGGCTCGGCGATCCAGCAAATTC
CGGAGCCGAGTAATTGGCATTATACCCGCCCGGTATGCTTCCTCTCGCTTCGAGGGTAAA
CCTCTTGTCAACATCCTCGGCAAGCCCATGATCCAGAGAACATGGGAAAGAGCGAAATTG
GCTGCATCATTGGATCATGTTGTTGTGGCGACAGATGATTATAAGATTGCTGAATGTTGT
AAAGGATTTGGTGCTGATGTTGTGATGACATCAGAATCTTGTCGAAATGGCACTGAGCGC
TGTAACGAAGCACTTCAAAAGATTGGGAAGCGGTATGATGTTGTTGTCAATATTCAGGGG
GACGAACCGCTCATAGAACCTGAAATAATAGATGGCATAGTCAAAGCTCTCCAGGATGCT
CCCGATGCAGTCTTCAGCACCGCAGTCACTTCCTTGAAGTCTGATGATGCTTTTGATCCA
AATCGTGTAAAATGTGTAATAGATAATCATGGCTATGCAATTTATTTTTCACGAGGACTT
GTACCATTCAACAAGTCCGGAAAAGTCAATCCTCAATTTCCATATTTACTTCATCTGGGG
ATTCAGAGTTTCGATACAGAGTTTCTCAAAATATATCCAGAGCTTCCACCGACTCCTCTT
CAACTGGAAGAAGATTTGGAACAACTGAAGGTCCTCGAAAATGGCTACAAGATGAAGGTG
ATAAAAGTTGACCATGAATGTCACGGTGTTGATGTTCCAGAAGACGTAGAGAAGATAGAG
AATTATATGCGGCAAAGGAACTTAAGT

>Unigene27999
ATGAGCTCCGTTTTCCAGCTCCACCGCAACCAATCCCTTCCGCTCTCCCAAGCTCAAACA
ACGTCGGAGCCCTCCGGTCCTGGAATCCGCCGCAGGCTTTCATCAATCTCACTCAATCTC

AAGATCCAGGCGCCCTCCTCTTCCCCCGCCGCCGCCGCCGCCGCATGGGCCAGCTTCCGT
CGTTCCAAGTCGGTGTCCTCGATTGGGGATTTCGCCGGCAGTTCGATCATAAAGTGGTGG
AATTGGGGATGGGGCTGGATCTTATCCCGGAAACCTACTTTCGCTTCAGATCTAGAAATG
AACGAAGAAGAAACGGCGGCTCTGGGCAGCCACAGCAAGGGCAGCTGGCGCCACGTGTAC
TACAAGGTATCCTCCCAGCTCCGCCGCAAGCTTCGCGGCTCCGACAATGTTGGACTCCCC
CAGACCTTCCGCTACGACTCCTCTAACTACCGAAAGAATTTCGACGACGGATCA

>Unigene408
ATGATGATGTTTGAGGATATGGGGTTCTGTGGTGATCTTGATTTCTTCTCCTCCTCGGGT
GGCATCAAGGAAGTTGAGGGCTGCCATGAGGCGGCGGAGGTGGAGGTCGAGGTGGAGGTG
GAGGCGGTGGCGGAGGACGATTACACGGACGAGGAGATCGACGTGGATGAGCTGGAGAGG
AGGATGTGGAGGGACAAGATGAGGCTGAAGAGGTTGAAGGAGATGAACAAGGAGAAAGAG
GGGGTTGATGCTGCCAAGCAACGGCAATCTCAGGAGCAAGCCAGGAGGAAGAAGATGTCT
AGAGCTCAAGATGGAATCTTGAAGTACATGTTGAAGATGATGGAGGTGTGCAAAGCTCAG
GGCTTCGTTTATGGCATCATCCCGGAGAAAGGGAAGCCGGTGAGCGGAGCATCCGATAAT
CTCCGGGAGTGGTGGAAGGATAAGGTCCGTTTCGATAGAAATGGACCGGCTGCTATAGCC
AAGTACCAGGCCGATAACTCCCTCCCGGGGAAGAACGAGGGGTGTAACCCGGTCGGGCCG
ACCCCTCACACCTTGCAGGAGCTCCAAGACACTACTCTTGGATCCCTGCTGTCTGCACTG
ATGCAGCACTGTGATCCGCCACAGAGGCGGTTCCCCCTGGAGAAGGGGGTCCCGCCTCCG
TGGTGGCCCACCGGGAAGGAGGAATGGTGGCATCAGTTGGGGTTACACAAGGATCAGGGG
CATCCACCTTACAAGAAGCCTCATGATCTGAAGAAGGCTTGGAAGGTGGGTGTTCTTACA
GCAGTGATCAAGCACATGTCTCCTGACATTGCCAAGATTCGGAAGCTGGTTAGACAATCC
AAGTGTTTGCAGGACAAGATGACTGCCAAGGAGAGTGCAACATGGCTGGCTATCATCAAT
CAGGAGGAGGCCTTAGCTCGGGAGCTTTACCCCGATCGCTGCCCGCCTCTGTCCTCGTCC
GGTGGCAGCGCCACCTTCGCGATGAACGACAGCAGTGAGTACGATGTAGACGTGAACGAT
CACGAGGAGTCGAATTTCGATGTGCAAGAGCAGAAACCAGCCACACTTGGTCTGTTGAAC
ATTGGTGGGATCAAGGATGAGGTGATCACCAACTTGGATTTCTCTAGGAAGAGAAAAGGG
GGGAATGAAGTGAACTTGATGATGGATCACAATAAGATATACACCTGCGAGTATCTTCAA
TGCCCTCACAGTGATCTTCGACATGGCTTTCAAGATCGAGCTTCAAGGGACAACCATCAG
ATGGCTTGCCCTTACACTCATTCCTCCCAATTCGCCGTCTCAAACTTCACCATCAACGAC
ATCAAGCCGATCGTCTTCCCTCAGTCGTTTATCCAGCCGAAGCCGGCTAGCCACACACCA
GCAGTACCACCACCACAACCCTTTGATTTATCAGGCCTTGGAGTACCAGAGGAAGGTCAA
AGGATGATCAATGAGCTCATGTCCTGTTATGACAACAACATCCTAGGAAACAAGAACACC
ACCAAGGAAGGCTACATTCACAGCCAAGGAATGGTGTTGGAGGGCAACATGTTCGATGAC
GACAACAACAACAACAACAGTAGCCACACACACAACGGTTCCATGTTCCGTTCGACGACG
GATCGGTTCGACCAGTGCAAGGTCATAAACACACCATTCAATCACAACACAAGTGACAAT
TTCCAGCTAATGTTCAGTTCTCCATTCAACATCTCTCCAGTTGATTACACACAGAATTTC
CATGGAGGTGCAAGGGATAACCTGCAAAAGCAGGATGTCTCAATCTGGTAC

>Unigene48122
ATGAGAGATGGAAACTTGAAGAAAAGTAAGCTTTCATGGCCTAAGACACTAGTCAAGAAA
TGGTTGAACATCCAGAGTAAGGATGAGGATTTTCATGCTGATGAATTCACTTATGCAGAT
GTTGATGAAGAATGGAGGAATAATTTCTCAGAGAGGGATTTATGCTCTATCAAGAAAAGC
AAAACAGAGCAATTGAATACTGAAAATCACGACTCTGTGCGAAGAAATAAGATTGGGTTC

GATTCCTCCCAAATTACAGATGTGCAGAACTATAGGATCTTTGTGGCAACTTGGAATGTG
GGTGGAAAGTCTCCAACGAGTCACTTGAATCTGGAGGATTGGCTAAACACTTTGCCTCCT
TCCGATGTTTATGTTCTTGGTTTTCAAGAAATTGTTCCTTTAAATGCTGGAAATGTTTTG
GGGACAGAAGATAATGGCCCAGCTAGGAAATGGCAAGCACTTATTCGAAAAACTCTGAAC
AATCTTTGTGGTAGTGGTGGTGGTGGTGGTGTTTGTCCTGTTCCATATCTGTCTCCAAAT
CCTGACCCATATGGTGAACTCTATGATGACTTTGAAGGGTCAAACAGAGAAAACGACTTG
TTCTTCCACCGGCACTCTGTGCATTCAATGAGCCGTAGCATGAGAATGGTGGATAGCAGC
ATTTCAATGCATCAACCCAGACTTGACCATCGTCTCAGTGTTTGTGATCGGGTTATCTAC
GGGCATCCAACTATGAGGTGGGATGCTGCTTCCTCTGATGATGAGGATGAACCAAATGAT
GCATTTTGTGCACAATCTTTTCCCATTGATGATGGAGACAAACATTCAGGGCAGTCAAGG
TATTGTTTGGTTGCAAGTAAGCAGATGGTCGGGCTATTTCTCACAGTGTGGATCAAGCGT
GATTTAAGAGACGCTGTTCGTGATTTGAAGGTGTCGTGTGTTGGCAGAGGGTTGATGGGT
TATCTTGGCAATAAAGGTTCAATTTCTATTAGCATGTATTTGCACCAAACAAGCTTTTGC
TTCATCTGTAGCCACTTGACATCTGGAGAGAAGGATGGAGACGAGCTGAGACGAAACTCC
GACGTGATGGAGATTCTGAGAAAAACAAGATTCCCTCAAGTTCATGCCGCAGGAGACGAG
AACAGCTCCCCTCAAACAATCCTTGATCACGATCGAATCATATGGCTCGGGGACTTGAAC
TACAGGATCGCCCTCAATTACCGGTATGCAAAGGCTCTTGTCGAGATGCACAACTGGAGA
GCCTTGTTAGAAAACGACCAGCTTACTATAGAGCAGAGGCACGGGCGTGTCTTTCCCGGA
TGGAACGAAGGGAAAATATACTTCCCTCCGACGTACAAGTATTCAAACAATTCCGACAGA
TATGCTGGTGAAAACATTCGCCATAAAGAGAAACGGAGAACTCCTGCTTGGTGTGACCGT
ATACTATGGCACGGCCGCGGTCTCCAACAACTCTCGTACGTTCGTGGGGAGTCCAGGTTT
TCAGACCACAGACCAGTGTATGGTGTATTTTTAGCTGAAGTCGAGTCGACAAAATGTAAC
CGTATCAAGAAAAGCATGAGCTATTCGAGCTCACGAGTTGAGATCGAAGAGCTCCTCCCA
TGCTCCCACGTCTACGGTCAACTGAATCTCTTC

>Unigene39432
ATGGGGCCGATCGTCAAGAGGAAGAAGAAGGGGAGGCCGGCGAGGGCAGATCCCGGTGCC
AGGGACCTCCCGTCGGCGGAGCGTGACCTCCGGAGAAGCCTCCGTCGCCGGAATGTGAAG
TACGTCTTCGACCTCGACGATTACTTCGACGAGGACGAGGTGTTCGCCGACGACGAGGAC
CAGCGACGGAGAGAGAAGAAGCTGAAGCTGCTACTGAAGCTCCAGGGCGGCGCCGATCCC
GAGTCAACTGGTCTCCACGCGCCTGCCAGCTCGGCGTCCTCGTCGGACGATGGTGGGAAG
CCGTCGAAGAAGCGGAAAATCGACGAAGAAATGGACGATGATGTGGATGACGATAATGAG
GAAGATAATTATAATTATGAAGACGATGAAGAGGTTAGAGATACAAAGCCAGAATCCAAA
GCTGATGACTCTCCACCAGGGACGCCGCCGTGTGGGCCACCGATGCCGGAGAAGAAGATT
CTGGAGTTAATTCTGGATAAACTTCAGAGGAAAGACATTTATGGTGTTTATGCAGAACCG
GTTGATCCAGAGGAGCTTCCGGATTACCACGATGTGATCGAGCATCCAATGGATTTTGCC
ACTGTGAGGAACAAGTTAAGAAATGATTCATATCCAACCTTTGAACATTTTGAGAGTGAT
GTCTTCCTCATTTGCTCAAATGCAATGAAATACAATGCACCAGATACCATATATTACAAA
CAGGCGCGCACCATCCAAGAGTTGGCGAGAAAGAAATTTCAAAAATTAAGGCTGAAAGCT
GAACACACGGAGAAAGAGATCAAACCCGAGCAGAAAACAAGGTCCGGCTCCCTTCCAAAA
AAGCCAATCAGAAGGTGTGGGAGCCGGACACTGCAAGAACCTGTTGGCTCCGATTTTTCA
TCTGGAGCCACTCTTGCCACAGTGGGAGATTTCCATAATGTTCCCAGTACACTCCAAGCT
GTTGGGTCCGAGAAAATGGGTGGTGCAGATGGGCTTGTTGAGGGAAGTTCTTTCTTGAAC
GATTACTGTTATGATAGGGTCGAAGAATCCCTGCCAGGAAAGGGTCCACTGTCTCGATTT

GGTAGGAGATCATTTGTTCAAGATGAAAACCGTCGTGCAACATATAACATTTCTTTGGCT
CTTCCTGTGGCCAGCTCAGAGTCTATATTCTCAACCTTTGATTGCGAAACCAAGCAGCTT
GTTCCTGTAGGACTTTATTCTGATCATTCATACGCCCGGAGTCTAGCTCGATTCGCTGCA
ACTCTTGGATCCGCTGCCTGGAAGGTTGCGTCTAAGAGGATCGAACAAGCACTACCTCAA
GGATTCAGGTTCGGTCAGGGTTGGGTTGGAGAGTATGAACCACTCCCAACTCCTGTACTA
ATGGTAGAAAATTGCTGTGTTAAAGAACCTCCGTTTTTAACAAAAGCAAAGCCTGTTGCT
AATCCTAAGAAGTTTGAGAAGACCCCAACAGTCCTGGATTCTTACAAGGATGATAGCCCA
CGTAGCGAGCCATTCCTGGGACAAAAGTTGCCGTTTCTTTGTCCCCCCGGAATCAGACCA
CCTTCTGCTTCTGTCGTCGCTACACAGCCGATCAGAGGGAACATTTCAGAGATGAACATG
AACCCATCCTACTTTTTGTCCTCCGGGATCAAACCTAGTAGCCCTCGTAGTAATCTCGGT
TATCATCATCAAAATCTTCAATCCAGGGCTGTGCATGAGTCTGAAAAGAAGGCTGTAAAG
CAGGTGGAATTGAATAGTCCTCCATCGTCGAATAATGTTGCATCTGATTCTTTTGGCAAA
AGACAGTTTCGGAAGAGTTCAGAAATGGAAGCCTCCAGGCCGATGGACTACTCGCCAAAG
AACACAAACCTCCCACGAACCGGATCGTTTAAAAGGCCCGATCATAGCGATGGGGTTGCT
ATGGTAGGAATGCCTAATGGAAAAGTCATGGGTAATAGAGTGGATAGTGACACTACAATG
CCTAGTTTGTCTTCTGATTCAGCAAAACCAGCCGGGTATGTCGAACAGGGTCAAGGTCTC
AATGACCCTGTCCAGATGATGAGAAAGATGTCTGAAAACACTCATAATCAACAAAAACCC
TCGAGGCGATCGCCAGCCAATGTTTCTCAAGTCATGCCTCCATCGCCATCCTTGAACAGC
AATGGATGGAATGGGAATGATCCGAACAATGCTGCTGCTGCTGCGCGAGCATGGATG
TCTGTAGGGGCGGGAGGGCTGAGACCTGTTGCTGAAAATGCAGCAAATCCGAACAAGAGC
CAAATGTACGGTGATCCAGTGTACAATTCGCCTCGGCAACAGTTTCGAGGAGAGTTCCCT
CATGGAGCTCCTCTCCATGCTTTCGTACCGCAAGGGCCAATACCTATGTTAGTGGGCAAT
CAAGTACAGTTTCAGAACCAGCGAATGGTGTTCCCAGGCCAGCTGGCGACCGCTGATTTG
TCGAGGTTCCAGCTGCAGCCGAATTGGCGGCCACAAATGCATTCGAGACAAAAACAGGAA
TCACTACCTCCAGACTTAAACATAGGCTTCCAAACATCCGGCTCCCCCGGACGGCCCTCT
TCCGGTGTGCTGGTCGATTCCCAGCAGCCCGATTTGGCTCTACAGCTC

>Unigene43199
ATGGGAAACTGCCAAGCCATTGATAATGCAACTCTTGTAATCCAACACCCTAATGGCAAA
GTGGACAACCTCTTCTTCCCTGTTCCTGCTTCTGAAATCATGAAGGCCAACCCTGGTCAC
TACGTTGCCCTCCTCCTCACCACCACCCTCTACTCTTCCACCGCTTCCGCCGCCGCCGCC
GCCGCCTCCAACAACAAGGACAACAAGAACCTCCCTCTTCGTGTCACTCGCATCAAACTT
CTCAGACCAACTGATACTCTTGTTCTTGGTCATGTCTACAGACTTGTCACCTCTCAAGAA
GTTATGAAGGGATTATTGGCAAAGAAGCAAGCCAAGATGCAGCAGCTGAAAAATGTAAAT
ATGAGAGAGAAGAAAATTTCTGATTTTGAAAAAAATGATGGAAAGAAGAGCAATAATCAG
GCAAAGCAAGAAAGGCACAGATCAAGAAACAACCCACAAGTAAACTCAGCTGCATCCACA
TCCAGAGCATGGCAACCAGCATTAAAAAGCATTTCTGAAGCTGCAAGC

>Unigene30075
ATGGGCTCTTCCCAATCAGCTCAAGTCCCCTCCTCTGACGAAGCAGAAGAAGAAGAAGAA
GACGACGACGTTGAGGACGAAGACGACGACGTTCAGCAGAACCTCACCCTGAACAGCACA
GATCATGTCAAGAAAATCCTCGAACAAGAGCCGGAGATGCTGCCGTGTCACGCCTCCGCG
TCGCCGCTGTCGCCGCAGCTCTCCGCCTACGGCACTCCCCGCACCGGACCTTCCATCAAG
GTGTGGGACCCTTACAATGTCCTCGCGCCGCCTCCGTCGCTGCCGCCGCTGACGCAGCAC

TTCCACCGCGGCTTCCCGGTGGCCCCCGCCGAAGACGATCGGGCGGTCACCGAAGTGTAC
TTGATCAGCCACGGCGAGTGCCACATGAATTTGAGGCCTGATTTGATCGCCGGGCGCTGC
CCCGATGCGGCGTTAACCTCCAATGGGAAACGACAAGCTCGTGCCTTGGCCGTGTTCTTG
AAGTCTCAGGGGGTGAGGTTTAACGCCGTTTACACTTCGCCGTTGGATCGGGTTCGGGCC
ACAGCGCAATCCGTTTGCCAGGAGCTGAATTTTGCAGAGGAACATGTACAAACATCCGAT
GCACTTCTCGAGATGAGCCAAGGTCACTGGGAAGGATGCCACAGGTCCGAAGTTTTCACC
CCCGAAACCACGAGTATAATGGAGAAATTCCAGCCTGATTTTTCACCACCATCCGGAGAA
TCACTCAGGCAAGTAGAGTTCCGGATGGTTCAGTTCCTAAACAGTACGGTCACGGCATTT
CCTGACAAATTTAGGTCTGATTTCTCCCCACCAGATCCTAGTGACAACCCAGCCTTCCAG
AACCATAGCTCTCACACCTTGCCAAACATGATCAATCACCGAGATGGGCCGTCTCTCGTA
CCTCCGAGCTGGGAGCTGCTACGCAGGCACCGGCAGGGACTCCCGAGGAAGAAATCGGGC
AAGAGCCGGCTCCAAATTGTGGCGACTACGGGTGATCACGAGGCCGACGATGAGATGTCG
CCCCGGGAACCCCATAATCAAGGACTAATCCGAGACATAAACGTCCGGACCGCCCCCTCG
CTCGTCACTTCTTGTATTGGGGTTTTCAGTCACTCCACACCGATCAAATGCCTTCTCACT
GGCATCCTCGGCTGCAGCCCGGTGATGTCGCAGAAGCTTTGCATCGAAGATTCTTCGGTC
ACCGTGCTGCAACGTTCTTTGAAATTCGGGTGGCAAATAAAGAGACTGAACGATACGTCA
CATCTTAGGCTTCTT

>Unigene24314
ATGTCATCACTAACTAAAATGGAAATCGTACCTCCACTACTGCTCCTCCTTCTCTGTACC
CTCCTCCACACCTCCGCCGCCGGACGCCGCGGCGGCATCGCCGTCTACTGGGGCCAGAAC
GGCAACGAGGGAACCCTCGCCGAAACCTGCGCCACCGGCAGATTCTCCTACGTCAACATC
GCCTTCCTCAACAAATTCGGCGGCGGCCAAACCCCCGAGCTCAACCTCGCCGGACACTGC
AATCCCGCCACCAACTCCTGCCGCGTCGTCTCCGACGGAATCCACGCCTGCCAGCGCCGC
GGCATCAAGGTCATGCTCTCAATCGGCGGCGGCATCGGAAACTACTCTCTCTCCTCCAGA
GAGGACTCCATAGGATTCTCCATCTACTTATGGAACAATTTCCTCGGCGGCGGCGGCGGC
GGATCTGCAATCCGCCCCCTCGGCGACGCCGTTTTGGACGGAATAGATCTGGATATCGAG
CTCGGATCTCCCCTGTTCTACGACGTTCTCGTGACGCATTTGAAATTGCTGAGCAGGAGG
CGGAGAAGAGTGTACGTCACCGGAGCGCCGCAGTGCCCGTTCCCCGATCGGTTGTTGGGG
GCGGCGCTTAATACGACGGCGTTCGATTACGTGTGGGTGCAGTTCTACAATAATCCGCCA
TGCCAGTACACTCCGGGGAACATTGAGAATTTGAAGAATTCGTGGGAGGTGTGGACGAAT
TCTGTTTACGCTGGGAAGATATTTTTAGGGCTTCCCGCGGCGCCGCAGGCTGCCGGAAGC
GGTTTTATTCCAGCGGATGTGGTGACGAGGGAGATTCTTCCGGTGATTAGGAGATCGAGG
AAGTATGGTGGAGTGATGCTGTGGTCGAAGTTTTGGGATGATCAAAGTGGTTATAGTACC
GCA

>Unigene30783
ATGTCTCAAACAAACTGGGAAGCTGACAAAATGTTGGACGTCTATATTCACGATTATTTA
GTGAAGAGAGATTTGAAGGCCACCGCTCAGGCTTTCCAAGCTGAAGGAAAAGTATCATCT
GACCCTGTTGCTATCGATGCTCCTGGTGGTTTTCTGTTTGAATGGTGGTCAGTGTTCTGG
GATATTTTTATTGCCAGGACTAATGAGAAGCACTCTGAAGTAGCTGCAACTTACATTGAG
CAGACTCAGCTAATGAAGGCAAGGGAGCAGCAACAACAGCAGCAGCCACCACAGTCACAA
CAGCAGCAGCAACAACAACAACAGCAACAAATGCAGATGCAGCAGATTTTATTGCAGAGG
CAGGCCGCCCATCAGCAGCAACAGCAACAACAGCAACAGCAACAACAACAGCAGCAGCAA

CAACAGCAACAGCAGCAGCAGCAACAACAACAGCAGCAACAACAACAGGCACATCAGCAG
CAGCAACGGCGTGAAGGTGGCCACCTCTTAAATGGCTCAGCCAATGGAATTGTTGGAAAC
GATCCTCTTATGCGACAGAATCCTGGTACTGCCAATGCTTTGGCGACGAAGATGTACGAA
GAGAAATTAAAGCTCCCTGTTCAGAGGGATTCTTTGGAAGATGCAGCTTTAAAGCAAAGA
TTTGGGGACAATGTTGGGCAGCTTTTGGATCCAAGTCATGCTTCAATCTTGAAATCAGCT
GCAGCAGCTGGCCAGCCATCTGGGCAAATGCTTCATGGTTCTGCTGGTGGAGTATCTCCC
CAAGTTCAAGCACGAAGCCAGCAATTTCCCGGGTCTTCACCAGACATTAAGACTGAGATG
AATCCTATCCTGAATCCAAGGGCTGCTGGTCCTGAAGGATCTTTGATTGGAATCCCTGGG
TCAAATCAAGGTGGTAACAATTTGACTTTGAAAGGATGGCCCCTGACGGGCTTCGATCAA
CTTCGTTCTGGGCTTCTGCAGCAGCCTAAGTCATTTATGCAGGGACCCCAGCCTTTTCAT
CAATTACAGATGCTAACGCCTCAGCACCAGCAGCAGCTTATGCTTGCCCAACAGAGTTTG
ACTTCCCCATCCGCCAATGAAGTAGAGAGCAGAAGGCTGAGAATGCTTCTTAATAGCCGA
AGTTTGTCAATGGGGAAAGATGGTCTCTCCAATTCAGTTGGTGATGTAGTTCCTAATATT
GGATCCCCCTTGCAAGCTGGTTGCCCGGTTATACCTCGAGCAGATCCTGAGATGTTGATG
AAGTTGAAAATCGCACAGATGCAGCAGCAGCAGCAGCAACAGCAGCAGAACAATAATCAA
ACACCACAGCAGCAGCTATCGCAACATGCTCTCTCTGGTCAGCAGCCTCAGAGTTCAAGT
CACAATCTCCAGCAAGATAAAATTATGGGTGCTGGCAGTGTAACTGGTGATGGAAGCATG
TCAAATTCATTTCGGGGAAATGAACAGGCTTCAAAAAACCAGACTGGAAGAAAGAGAAAA
CAGCCTGCATCTTCTGGTCCTGCTAACAGCTCGGGGACTGCGAACACAGCCGGTCCATCT
CCAAGTTCGGCTCCCTCAACACCCTCAACACATACGCCTGGAGATGTGATGTCAATGCCT
GCCTTGCCTCACAGTGGCAGTTCTTCGAAACCTATGATGATGTTTGGACCTGATAACACT
GGTACTCTGACATCACCATCAAACCAGTTGTGGGATGACAAAGATCTTGTACAAACTGAT
ATGGATCGTTTTGTGGATGATGTTGAAGATAATGTAGAGTCTTTTTTATCACATGATGAT
GCTGACCCCCGAGATACAGTTGGTCGTTGTATGGATGTCAGCAAGGGTTTTACATTTACG
GAAGTGAACTCTGTTCGTGCTAGTTCTAGTAAAGTCGTGTGTTGCCATTTCTCATCAGAT
GGAAAGTTGCTTGCCAGTGGTGGTCATGATAAAAAGGCTGTTTTATGGTACACAGATAAT
TTAAAACCTAAAACAACTCTTGAGGAACACACATCACTTATTACTGATGTTCGTTTCAGC
CCTAGCATGGCTCGCCTTGCCACATCATCCTTCGATAAAACTGTCAGAGTGTGGGATGTC
GATAATCCTTCGTATTCTCTTCGGACCTTTACTGGACATTCTGCTGGTGTAATGTCATTG
GACTTCCATCCCAACAAGGATGACCTCATCTGCTCATGTGATGGTGATGGTGAGATAAGA
TACTGGAGTATCACTAATGGAAGCTGCCAAAGAGTTTTCAAGGGTGGAACGACCCAAGTG
AGATTCCAACCACGTCTTGGAAGGTATCTTGCAGCAGCAGCTGAAAACGTTGTGTCAATA
CTGGATGCGGAGACGCAAGCCTGTCGACATTCATTAAAGGGACATACGAAACATATTCAC
TCCGTTTGCTGGGACCCTTCTGGTGAGCTCCTAGCATCTGTTAGTGAAGATTCAGTGAGG
GTGTGGACGTTGAGATCCGGAAGTGAAGGCGAATGTTTACACAAGCTTAGTTGCAATGGG
AACAAATTCCATTCTTGCGTTTTCCACCCTACGTATTCTTCATTGCTTGTCATTGGTTGT
TATCAGTCGCTGGAGCTGTGGAACATGAGTGAGAACAAGACGATGACACTCTCTGCGCAC
GAAGGATTGATTGCTTCGTTGGCTGTATCGACGGTGGCGGGCGTGGTGGCGTCTGCTAGT
CACGATAAGATTGTTAAGCTGTGGAAG

>Unigene27774
ATGGCGCTTCAGTTGTGGGAAACACTGAAAGAATCTATAACAGTCTACACAGGCCTCTCT
CCGGCTACTTTCTTCACTGTTCTTGCCTTGGGCCTCACCCTTTACTACTTTGTTTCCAAT
TTGTTTGGTTCCTCTGATGATGGACATGTCCAGCACACTTCCAGAAGCTTCGAGGAGCAC

GTGGAGCCTCTGCCTCCTCCGGTTCAGATCGGCGAGATCTCCGCTTATGAATTGAAGCAA
TACGATGGTGCTGATCCGAAGAAGCCTCTGCTTATGGCTATCAAGGGTCAGATCTATGAT
GTGTCGCAGAGCAGGATGTTTTACGGACCGGGTGGACCTTATGCATTGTTTGCTGGAAAG
GACGCGAGTAGAGCGCTAGCAAAGATGTCCTTCGAGGACAAAGATCTGAACGGTGATCTG
ACTGGCCTGGGCGTGTTTGAGCTTGAAGCTCTCCAAGATTGGGAGTACAAATTCATGAGC
AAATATGTCAAGGTTGGAACTGTGAAGGCGGTCGTACCAGTTACCGATGGTGCGGCTGAA
GGCCAAGCGGACGTTGCTACTGAAAGCGCTGCTGCTGCTGCTGATAAGCCTGCAGAA
GGTGCTGAGGGGGATGTTCTAAAACCCGCGGAAGAAGGCCCATCAGAGAATGCTGCTAAG
GTTACTGAGGAAACCACACCAGCTAGTGATGCGGACAAAAAG
